# Supplementary material for: Improving uptake of Fracture Prevention drug treatments: a protocol for Development of a consultation intervention (iFraP-D)
Source: BMJ Open. 2021 Aug 18;11(8):e048811. doi: 10.1136/bmjopen-2021-048811 (PMC8375717; doi:10.1136/bmjopen-2021-048811)
Supplement: Supplementary data [file bmjopen-2021-048811supp001.pdf]

Supplementary Table 1. Eligibility criteria for osteoporosis guideline evidence synthesis

Inclusion criteria	
**Population**	Patients at risk of osteoporosis or with fragility fractures
**Interventions**	Clinician-patient consultations to assess risk and initiate/recommend treatment for fracture prevention
**Outcomes**	Assessment<br>Explanations<br>Decision-making
**Setting**	Relevant to UK primary or secondary care (e.g. UK, European or international)
**Date**	Those guidelines that have been developed, reviewed or revised within the past 10 years will be used (2009 to Feb 2019)

Exclusion criteria

Guidelines relating to:

- managing patients already on fracture prevention treatments
- disease specific conditions (e.g. screening for osteoporosis in inflammatory bowel disease)
- patients with one specific fracture site (e.g. hip fracture)
- steroid-induced osteoporosis
- specific countries other than UK
- fracture assessment and management

Guidelines related to pre-clinical and animal studies
Withdrawn guidance
The original research manuscripts underpinning the guidelines
Studies not in the English language.
