# Supplementary material for: Improving uptake of Fracture Prevention drug treatments: a protocol for Development of a consultation intervention (iFraP-D)
Source: BMJ Open. 2021 Aug 18;11(8):e048811. doi: 10.1136/bmjopen-2021-048811 (PMC8375717; doi:10.1136/bmjopen-2021-048811)
Supplement: Author's manuscript [file bmjopen-2021-048811.draft_revisions.pdf]

BMJ Open is committed to open peer review. As part of this commitment we make the peer review history of every article we publish publicly available.

When an article is published we post the peer reviewers' comments and the authors' responses online. We also post the versions of the paper that were used during peer review. These are the versions that the peer review comments apply to.

The versions of the paper that follow are the versions that were submitted during the peer review process. They are not the versions of record or the final published versions. They should not be cited or distributed as the published version of this manuscript.

BMJ Open is an open access journal and the full, final, typeset and author-corrected version of record of the manuscript is available on our site with no access controls, subscription charges or pay-per-view fees (http://bmjopen.bmj.com).

If you have any questions on BMJ Open's open peer review process please email info.bmjopen@bmj.com

BMJ Open

Improving uptake of Fracture Prevention drug treatments: a protocol for Development of a consultation intervention (iFraP-D)

Journal:	*BMJ Open*
Manuscript ID	bmjopen-2021-048811
Article Type:	Protocol
Date Submitted by the Author:	11-Jan-2021

Complete List of Authors:	Paskins, Zoe; Keele University, School of Medicine; Haywood Hospital, Academic Rheumatology Centre<br>Bullock, Laurna; Keele University, School of Medicine<br>Crawford-Manning, Fay; Keele University, School of Medicine; Haywood Hospital, Academic Rheumatology Centre<br>Cottrell, Elizabeth ; Keele University, School of Medicine<br>Fleming, Jane; University of Cambridge, Cambridge Public Health; Addenbrooke's Hospital, Cambridge University Hospitals NHS Trust<br>Leyland, Sarah; Royal Osteoporosis Society<br>Edwards, John; Keele University, School of Medicine<br>Clark, Emma; University of Bristol, Bristol Medical School<br>Thomas, Simon; Keele University, School of Pharmacy and Bioengineering<br>Chapman, Stephen; Keele University, School of Pharmacy and Bioengineering<br>Ryan, Sarah; Keele University, School of Medicine & School of Nursing and Midwifery; Haywood Hospital, Academic Rheumatology Centre<br>Lefroy, JE; Keele University, School of Medicine<br>Gidlow, Christopher; Staffordshire University, Centre for Health and Development<br>Iglesias, C; University of York, Department of Health Sciences; Aalborg Universitet, Danish Centre for Healthcare Improvements<br>Protheroe, Joanne; Keele University, School of Medicine<br>Horne, Robert; University College London, Centre for Behavioural Medicine, UCL School of Pharmacy<br>O'Neill, Terence; University of Manchester, Centre for Epidemiology Versus Arthritis; Manchester University NHS Foundation Trust, NIHR Manchester Biomedical Research Centre<br>Mallen, Christian; Keele University, School of Medicine<br>Jinks, Clare; Keele University, School of Medicine

Keywords:	RHEUMATOLOGY, Musculoskeletal disorders < ORTHOPAEDIC & TRAUMA SURGERY, QUALITATIVE RESEARCH

I, the Submitting Author has the right to grant and does grant on behalf of all authors of the Work (as defined in the below author licence), an exclusive licence and/or a non-exclusive licence for contributions from authors who are: i) UK Crown employees; ii) where BMJ has agreed a CC-BY licence shall apply, and/or iii) in accordance with the terms applicable for US Federal Government officers or employees acting as part of their official duties; on a worldwide, perpetual, irrevocable, royalty-free basis to BMJ Publishing Group Ltd ("BMJ") its licensees and where the relevant Journal is co-owned by BMJ to the co-owners of the Journal, to publish the Work in this journal and any other BMJ products and to exploit all rights, as set out in our licence.

The Submitting Author accepts and understands that any supply made under these terms is made by BMJ to the Submitting Author unless you are acting as an employee on behalf of your employer or a postgraduate student of an affiliated institution which is paying any applicable article publishing charge ("APC") for Open Access articles. Where the Submitting Author wishes to make the Work available on an Open Access basis (and intends to pay the relevant APC), the terms of reuse of such Open Access shall be governed by a Creative Commons licence – details of these licences and which Creative Commons licence will apply to this Work are set out in our licence referred to above.

Other than as permitted in any relevant BMJ Author's Self Archiving Policies, I confirm this Work has not been accepted for publication elsewhere, is not being considered for publication elsewhere and does not duplicate material already published. I confirm all authors consent to publication of this Work and authorise the granting of this licence.

For peer review only

Title

Improving uptake of Fracture Prevention drug treatments: a protocol for Development of a consultation intervention (iFraP-D)

Authors

Dr Zoe Paskins. School of Medicine, Keele University & Haywood Academic Rheumatology Centre, Staffordshire and Stoke-on-Trent Partnership Trust, Stoke-on-Trent, Staffordshire, UK. ORCID: 0000-0002-7783-2986

Dr Laurna Bullock. School of Medicine, Keele University, Staffordshire, UK. ORCID: 0000-0002-4193-1835

Dr Fay Crawford-Manning. School of Medicine, Keele University & Haywood Academic Rheumatology Centre, Staffordshire and Stoke-on-Trent Partnership Trust, Stoke-on-Trent, Staffordshire, UK. ORCID: 0000-0002-9768-1695

Dr Elizabeth Cottrell. School of Medicine, Keele University, Staffordshire, UK. ORCID: 0000-0002-5757-1854

Dr Jane Fleming. Cambridge Public Health, University of Cambridge & Addenbrooke's Hospital Fracture Liaison Service, Cambridge University Hospitals NHS Trust, Cambridge, UK. ORCID: 0000-0002-8127-2061

Sarah Leyland. Royal Osteoporosis Society, Bath, UK

Dr John J Edwards. School of Medicine, Keele University, Staffordshire, UK. ORCID: 0000-0003-0432-7783

Dr Emma M Clark. Bristol Medical School, Faculty of Health Sciences, University of Bristol, Bristol, UK. ORCID 0000-0001-8332-9052

Simon Thomas. School of Pharmacy and Bioengineering, Keele University, Staffordshire, UK

Prof Stephen Chapman. School of Pharmacy and Bioengineering, Keele University, Staffordshire, UK

Prof Sarah Ryan. School of Medicine & School of Nursing and Midwifery, Keele University & Haywood Academic Rheumatology Centre, Staffordshire and Stoke-on-Trent Partnership Trust, Stoke-on-Trent, Staffordshire, UK

Dr Janet Lefroy. School of Medicine, Keele University, Staffordshire, UK. ORCID: 0000-0002-2662-1919

Prof Christopher Gidlow. Centre for Health and Development, Staffordshire University, Stoke-on-Trent, Staffordshire, UK. ORCID: 0000-0003-4990-4572

Dr Cynthia P Iglesias. Department of Health Sciences, University of York, York, UK & Danish Centre for Healthcare Improvements (CHI), Aalborg University, Denmark. ORCID: 0000-0002-3426-0930

Prof Joanne Protheroe. School of Medicine, Keele University, Staffordshire, UK. ORCID: 0000-0002-9608-1487

Prof Robert Horne. Centre for Behavioural Medicine, UCL School of Pharmacy, University College London, London, UK. ORCID: 0000-0002-3068-8438

For peer review only

Prof Terence W O'Neill. Centre for Epidemiology Versus Arthritis, University of Manchester & NIHR Manchester Biomedical Research Centre, Manchester University NHS Foundation Trust, Manchester, UK. ORCID: 0000-0002-8896-4677

Prof Christian Mallen, School of Medicine, Keele University, Staffordshire, UK. ORCIDL 0000-0002-2677-1028

Prof Clare Jinks, School of Medicine, Keele University, Staffordshire, UK. ORCID: 0000-0002-3407-2446

Correspondence:

Zoe Paskins z.paskins@keele.ac.uk

Tel 000 44 1782 733975 Fax: 00 44 1782 734719

Word count: 5617

For peer review only

Abstract

Introduction

Prevention of fragility fractures, a source of significant economic and personal burden, is hindered by poor uptake of fracture prevention medicines. Enhancing communication of scientific evidence and elicitation of patient medication related beliefs has the potential to increase patient commitment to treatment. The iFraP (**I**mproving uptake of **Fra**cture **P**revention drug treatments) programme aims to develop and evaluate a theoretically informed, complex intervention consisting of a computerised web-based decision support tool, training package and information resources, to facilitate informed decision-making about fracture prevention treatment, with a long-term aim of improving informed treatment adherence. This protocol focuses on the iFraP **D**evelopment (iFraP-D) work.

Methods and analysis

The approach to iFraP development is informed by the Medical Research Council complex intervention development and evaluation framework and the three-step implementation of change model. The context for the study is UK Fracture Liaison Services (FLS), which enact secondary fracture prevention. An evidence synthesis of clinical guidelines and Delphi exercise will be conducted to identify content for the intervention. Focus groups with patients, FLS clinicians and General Practitioners and a usual care survey will facilitate understanding of current practice, and investigate barriers and facilitators to change. Design of the iFraP intervention will be informed by decision aid development standards and theories of implementation, behaviour change, acceptability and medicines adherence. The principles of co-design will underpin all elements of the study through a dedicated iFraP Community of Practice including key stakeholders and Patient Advisory Group. In-practice testing of the prototype intervention will inform revisions ready for further testing in a subsequent pilot and feasibility randomised trial.

Ethics and dissemination

Ethical approval was obtained from North West - Greater Manchester West Research Ethics Committee (19/NW/0559). Dissemination and knowledge mobilisation will be facilitated through national bodies and networks, publications and presentations.

Registration

Research Registry (identifying number: researchregistry5041)

Article Summary

Strengths and limitations of this study

- A robust intervention development process will incorporate multiple sources of evidence, informed by the Medical Research Council framework for developing complex interventions and a three step model of change.
- A comprehensive logic model and use of the Necessity Concerns Framework, provides a theoretical basis for enhancing informed adherence.
- Collaboration with patients and clinicians, using the principles of co-design, and use of the Theoretical Framework of Acceptability to analyse qualitative data will enable us to iteratively develop an intervention that is relevant and acceptable to users.

For peer review only

- iFraP will be designed to address barriers to implementation from the outset, through the use of Normalisation Process Theory, Theoretical Domains Framework, and COM-B, and will produce evidence on barriers and facilitators to implementation.
- The research will be conducted in the UK, with in-practice testing conducted at one site; it is possible that barriers and facilitators to change, and the relevance of our intervention may vary across FLS sites, different contexts (e.g. in primary care) and geographical locations (nationally and internationally).

For peer review only

INTRODUCTION

In the UK, three million people are estimated to have osteoporosis,[1] contributing to over 500,000 fragility fractures (fractures resulting from low trauma) per year, costing an estimated £4.4 billion per annum.[2] Fragility fractures can be devastating, sometimes resulting in loss of independence and mortality.[3] Hip fractures alone account for 85,000 unplanned hospital admissions and 1.8 million bed-days in the UK per year.[4] Evidence-based treatments, such as bisphosphonates, are recommended by the National Institute for Health and Care Excellence (NICE) for patients with osteoporosis and/or a high fracture risk. They are inexpensive, cost-effective, readily available and reduce fracture risk by 20-70% (depending on fracture site).[5] Despite this, a treatment gap exists. Up to 80% of patients who experience a fragility fracture do not receive medication in the year following fracture,[6] 25% of people who are offered medication decline it (non-initiation),[7] and among those who do start bisphosphonates, few persist for long enough for it to be effective, with adherence estimated at 16-60% at one year.[8] Closing this treatment gap may prevent at least 20,000 hip fractures annually in the UK.[4]

Patient reasons for non-initiation and non-persistence of oral bisphosphonates (the mainstay of osteoporosis treatment) are complex and include: perceptions that drugs are not effective, not necessary and/or not safe; limited understanding of the consequences of non-treatment; and, concerns about perceived or experienced side effects.[9,10] Despite national osteoporosis guidance recommending the provision of information as a core component of management,[11] patients report that osteoporosis information provided in consultations is often not easy to understand.[12] Some primary care clinicians believe that bisphosphonates are not safe, effective or necessary.[13] The more recent shift to base treatment recommendations on fracture risk rather than bone density readings,[14] is not without challenge: patients struggle to understand fracture risk assessments[15] and frequently underestimate their risk of fracture.[16] This suggests unmet health literacy needs and patients have identified improving access to information from health professionals as the number one patient priority for osteoporosis research.[17]

Patients ultimately decide whether to start and continue taking medication, but this decision-making is influenced by the clinician-patient interaction. In order to decide to start and persist with medication, patients need to believe that recommended drug treatment is necessary, relevant, safe and practicable. Effective communication that enables patients to understand complex medical terms and concepts in lay terms, increases patient satisfaction, facilitates participation in the consultation, promotes trust[18] and may increase patients' commitment to medication.[19] This highlights the relevance of promoting and supporting effective communication between clinician and patients, and suggests that improving communication of the harms and benefits of osteoporosis medications may be beneficial in reducing the treatment gap.

Decision aids (DAs) include numerical estimates of risk/benefit. They can facilitate improved risk communication and support patient decision-making before or during health care consultations.[20,21] When used across a range of conditions, DAs can: increase patient knowledge, reduce decisional conflict, increase patient participation in decision-making and improve the accuracy of risk perception.[20] A recent Cochrane review reported high-quality evidence that DAs, across a range of conditions, increase patient knowledge and reduce decisional conflict, and moderate-quality evidence that DAs increase patient participation in decision-making and improve the accuracy of risk perception.[20] Increases in knowledge and informed choice were also reported in studies where health literacy needs were addressed.[22] Evidence from pooled analyses of studies where there was no equipoise

For peer review only

(meaning that DAs were used to give information about recommended drug treatments, rather than to choose between treatment options with perceived equal benefits), has indicated that DAs improve treatment initiation rates.[20] In osteoporosis, use of DAs can increase accuracy of risk perception and shared decision-making. However, existing osteoporosis DAs fail to comprehensively meet international quality standards and patient needs,[23] underlining the requirement for further development.

DAs are sometimes called Decision Support Tools (DSTs), particularly when tools also contain clinician decision support. DSTs can also support clinicians to follow evidence-based practice and have been shown to improve adherence to evidence-based guidelines.[24,25]

The iFraP (**I**mproving uptake of **Fra**cture **P**revention drug treatments) programme, aims to develop and evaluate a theoretically informed, complex intervention consisting of a computerised (C)DST, training package and information resources, to facilitate informed decision-making about fracture prevention treatment, with a long-term aim of improving informed treatment adherence. This protocol is for the first study within the iFraP programme of work focusing on the **D**evelopment of the new consultation intervention, referred to as iFraP-**D**.

For peer review only

STUDY PROTOCOL

Aims and Objectives

The iFraP intervention development study (iFraP-D) has three overarching objectives, which are to:

1. Develop core content for a new consultation intervention (iFraP) based on theory, published guidance, systematic review evidence, a Delphi consensus exercise and stakeholder engagement
2. Design a prototype CDST using published guidance and with stakeholder engagement
3. Use qualitative methods and stakeholder engagement to:
    a. Investigate current practice, and the barriers and facilitators to, communicating fracture risk and treatment benefits/harms and to delivering the iFraP intervention (CDST and training package), including the training needs of clinicians
    b. Co-design the components of the iFraP intervention and associated training package for clinicians
    c. Plan the integration of components in the new iFraP consultation intervention
    d. Conduct cycles of in-practice testing to determine if the iFraP intervention is meeting its objectives, with subsequent refinement of the intervention.

Overview of context and intervention

The proposed iFraP consultation intervention will be primarily designed for Fracture Liaison Services (FLSs). These are usually nurse-led, and address secondary fracture prevention by: systematically identifying patients with fragility fractures; assessing the patient's bone health, risk of falls and future fracture; and providing treatment recommendations to the patient and primary care. Clinical standards for FLS also recommend follow-up consultations, three and 12 months post-fracture, to support medicines adherence.[26] Within the context of FLS, all consultations are concerned with future fracture prevention, which will maximise efficiency of recruitment and testing. The multidisciplinary study team will allow the relevance of the intervention for use in other primary and secondary care settings in which fracture prevention treatments are recommended to patients, to be considered.

The iFraP consultation intervention will support clinicians to:

- Make decisions about who is eligible for fracture prevention treatment, using a CDST to operationalise existing clinical guidelines
- In patients in whom fracture prevention treatment is indicated:
    - Communicate the risks and benefits of fracture prevention treatment, including individualised fracture risk, using a CDST, adopting universal precautions for low health literacy
    - Elicit patients' understanding, and concerns about fracture prevention treatment
    - Assess readiness to initiate fracture prevention treatments and facilitate behaviour change.

For peer review only

  - Communicate consultation outcomes with the patients' primary care provider and facilitate consistent information provision across primary and secondary care

The iFraP consultation intervention will be delivered within FLS consultations and will include:

- A CDST to communicate individual fracture risk. This will include clinician decision-support and a patient-facing DA. It will be dynamic, interactive and tailored to risks and needs of the patient. It will incorporate fracture risk (calculated in external systems (e.g. FRAX)), an indicator for clinicians of whether treatment is recommended, a pictorial presentation of individualised fracture risk, fracture risk with medication (to show benefits of treatment), and possible treatment harms. The CDST will be used by trained clinicians in a model (face-to-face or remote) consultation with patients.
- Clinician training in delivering the consultation intervention, and supporting delivery of follow-up consultations. This will encompass a prioritised list of key tasks for the clinician (both information giving and eliciting) to undertake. Clinicians will be coached in listening skills, shared decision-making skills and universal precautions for health literacy.
- Information resources (paper and online) for the patient and GP to refer to after the initial or follow-up consultation, including a printout from the CDST.

Theoretical frameworks underpinning iFraP development

The long-term aim of the iFraP intervention is to improve fracture prevention treatment uptake, thereby preventing future fragility fractures. The intervention is targeted for use by clinicians together with the patient. This will empower clinicians to support patient behaviour change, and support clinicians with the skills to elicit and address patient perceptions and practicalities related to treatment adherence. The Necessity-Concerns Framework (NCF) will be used as an overarching theoretical framework when designing the iFraP intervention to understand patient's attitudes and beliefs underpinning treatment non-adherence.[27] The NCF suggests that medication adherence is influenced by implicit judgements of personal need for the treatment (necessity beliefs) and concerns about the potential consequences of treatment.[27]

To optimise iFraP in terms of acceptability to clinicians and patients, we will use the Theoretical Framework of Acceptability (TFA) [28] as an overarching framework to inform iFraP intervention design. The TFA consists of seven constructs of acceptability of healthcare interventions (affective attitude, burden, intervention coherence, ethicality, perceived expectations, opportunity cost, and self-efficacy). These constructs will inform data collection and analysis.

The implementation of iFraP is informed by the COM-B model of behaviour change [29] and the complementary Theoretical Domains Framework (TDF)[30] which aim to simplify and integrate a range of behaviour change theories. The TDF identifies 14 related domains of influence on behaviour. In iFraP-D, data collection will be informed by domains within the TDF (e.g. knowledge, beliefs about capabilities, skills, and goals). This will identify potential barriers and enablers for clinicians to implement iFraP ahead of a formal feasibility study. Normalisation Process Theory (NPT)[31] will underpin the investigation of the dynamics of implementing, embedding, and integrating iFraP, in order to identify potential process problems related to implementing iFraP in the next phase of feasibility testing. NPT ensures consideration of potential structural problems about the integration of iFraP into existing services.

For peer review only

The specific programme theory for the iFraP-D study is detailed in the study logic model (Figure 1). This initial logic model may undergo iterative changes throughout the iFraP programme. Throughout development, a health economist will define the decision problem enabling identification of the different elements of treatment effect potentially associated with the iFraP intervention and directly informing the data collection strategy, including primary and secondary outcomes, for the subsequent pilot and feasibility trial that will follow iFraP-D.

Approach to iFraP intervention development

In line with key principles of intervention development, our approach will be iterative, open to change and forward looking to future evaluation and implementation.[32] The Medical Research Council guidance on complex intervention development will be used as an overall framework,[33] within which we will specifically use a three-step implementation of change model.[34] This approach incorporates three pragmatic questions with three steps, each linked to iFraP study objectives outlined previously:

- **Step 1**: ‘Where do we want to be?’ Make a concrete proposal for change, and develop the content and format of the consultation intervention (objective 1).
- **Step 2:** ‘Where are we now?’ - Understand the current clinical context including barriers and facilitators to change (objective 3a).

  Step 3: ‘How do we get there’ – Develop a strategy to change behaviours, by designing and refining the prototype into a draft intervention, and field-testing (objective 2, 3b, 3c and 3d).

To answer the above questions we will take actions common to different intervention development approaches;[35,36] drawing upon theory, empirical evidence from the evidence synthesis, Delphi survey, qualitative focus groups and in-practice testing, stakeholder engagement, and guidance for the development of DAs.[37,38] We will adopt an informed design to iFraP design decision-making.[32,39] We will frequently engage with stakeholders as part of our Community of Practice (CoP) and Patient Advisory Groups (PAG) to discuss ideas generated by the study team and research findings.[39] The informed design will facilitate stakeholder involvement in iFraP intervention content and design decisions,[39] with final design decision-making made and documented by the Study Management Group [32] supported by the APEASE framework where appropriate.[40]

Each step and linked study objective are outlined in turn throughout this protocol. The inter-relation between the three steps and methods of data collection is displayed in Figure 2.

Co-design of the iFraP intervention

Community of Practice (CoP)

CoPs bring together expertise with a common concern or interest, with the aim of improving and learning to do better through regular group interaction.[41] We will bring together relevant expertise (FLS clinicians, GPs, osteoporosis specialists, commissioning representatives, patients (with experience of fracture prevention treatment, supported by a PPIE worker)), representatives from the Royal Osteoporosis Society (ROS) and Health Literacy UK and a behaviour change expert) in a stakeholder group that adopts a CoP approach.[41] The CoP will provide their views on the iFraP intervention content, and discuss the findings from the evidence synthesis, Delphi study, qualitative focus groups and in-practice testing.

Patient and Public Involvement and Engagement (PPIE)

For peer review only

The osteoporosis Research User Group (RUG) at Keele University comprises patients with experience of osteoporosis and/or fragility fractures, or carers for such patients. These RUG members had substantial involvement in a previous study to identify patient and public priorities for research in osteoporosis, which provided the starting point for iFraP.[17] Furthermore, the study specific PAG informed and agreed how PPIE members will be involved throughout the iFraP programme at the outset. In addition to PAG members participating in the Study Management Group, Award Steering Committee meetings, and CoP meetings, approximately six study specific PAG meetings will be convened throughout the iFraP research cycle. PAG meetings will: i) facilitate design, analysis and interpretation of the Delphi study (including how best to recruit and explain the Delphi study to patients, piloting the survey, and contributing to analysis), qualitative focus groups and in-practice testing (by informing recruitment, co-designing topic guide content and supporting analysis and interpretation of findings); ii) co-design visual components of the CDST; iii) informally test the CDST prototype before in-practice testing; and, iv) provide advice about dissemination to the wider public, including what results to share, when, and in what format. Additional information regarding PPIE is detailed throughout this protocol where appropriate. The GRIPP2 guidelines will be used in future dissemination to guide PPIE reporting.[42]

METHODS AND ANALYSIS

Step 1: Developing content and format of iFraP

In order to inform the content of the model consultation and the CDST components (overarching objective 1) of the iFraP consultation intervention, an evidence synthesis will be conducted of clinical guidelines, followed by consultation with stakeholders and a Delphi study with patients/carers and clinicians.

Evidence synthesis and stakeholder consultation

Search strategy - To identify osteoporosis clinical guidelines, the NHS Evidence electronic database will be searched using keywords ‘fragility fracture’ and ‘osteoporosis’ and ‘guidelines’ for studies that fulfilled the eligibility criteria (Supplementary Table 1). The search will be filtered to include guidance, quality indicators, and policy and strategy. Guidelines that were developed, reviewed or revised within the 10 years prior to search inception will be used. Generic NICE guidance relating to conducting the consultation will also be included.

Patient information resources will be identified using a Google search. Leaflets and webpages will be selected because they are easily available (e.g. on the internet, through patient organisations or places that people with osteoporosis might visit such as pharmacies). They will come from three different types of source: health care providers (UK National Health Service), charitable and voluntary organisations, and the medical profession.

Selection process - Eligible guidelines will be selected on title first by one reviewer (ZP). Full texts will be retrieved and assessed if the title, summary or abstract provides insufficient information.

Eligible patient information resources will be selected by two reviewers. A pragmatic and purposive sample of resources that are most commonly accessed and represent the three groups of information providers will be selected.[43]

Quality appraisal - The quality of the included guidelines will be appraised using the AGREE II tool.[44] Guidelines that score 75% or above will be tagged as high quality (in line with examples given by the AGREE II developers); this quality score will inform discussion in the stakeholder groups about the relevance of recommendations.

The overall quality of each patient information resource will be assessed using a modified International Patient Decision Aid Standard (m-IPDAS).[37] The m-IPDAS assess patient information based on 32 items across 8 categories of: content, bias, detail, probabilities, accuracy, decision-making, conflict of interest, structure and layout, and reliability.

Data extraction - Statements and recommendations from included guidelines that are relevant to tasks for the clinician in the consultation will be extracted and grouped into steps of phases of the consultation. Text from patient information resources will be extracted relating to descriptions of osteoporosis and osteoporosis drug treatment using a bespoke data extraction form in excel.

Analysis - A narrative summary will report the findings of the guideline evidence synthesis, including textual description of guidelines, tabulation of recommendations and exploration of relationships between and within guidelines.

Extracted data from the patient information will be thematically analysed independently by two members of the study team using the five domains of Leventhal's Common-Sense Model of Disease as a deductive framework.[45] Discussions will check the consistency of coding against the framework.

Stakeholder consultation - Evidence synthesis findings will be presented to the CoP for reflection on the findings and discussion of any inconsistencies, and discussion of how clinician tasks map to stages of the FLS consultation. The synthesis output (informed by the CoP) will form the basis of the Delphi.

Delphi Study

Design - Three rounds of a modified Delphi consensus study [46] will be conducted. The Delphi approach is described as 'modified', as participants will be presented with a list of statements to consider rather than generating their own list. However, in the first round, they will also have the opportunity to suggest additional statements for the consultation in free-text boxes. The aim of the Delphi is not only to make decisions about inclusion and exclusion but also, for included statements, to ascertain essential and optional consultation content. The same survey will be developed for all participants (both clinical and patient/carer) to develop a common, understandable language. Survey participants will be presented with clinical vignettes and a list of potential content for the model consultation. The content will be derived from the evidence synthesis, informed by the CoP, and written in partnership with PAG members.

Participants - Patients with osteoporosis and/or fragility fractures and/or their carers will be recruited through the ROS supporters' network. Clinicians who have experience of consulting with patients, where fracture risk is calculated and fracture prevention drug treatments are recommended, will be invited to participate through ROS clinician mailing lists and the study team's clinical networks. Clinicians will be multidisciplinary; from primary and secondary care and academic settings. We aim to invite up to 400 participants, anticipating a 40% initial response rate, and subsequent drop out at Rounds 2 and 3, with the aim of achieving a final sample of 15 patients/carers and 15 clinicians.

Data collection - In Round 1, statements will be presented that relate to tasks of the consultation, to include clinician decision-making tasks and considerations, eliciting information, giving information, example explanations and hypothetical use of the CDST, for example, 'the patient should be verbally informed of their individualised fracture risk', and 'the patient should be shown a picture of their individualised fracture risk'. Statements will be

For peer review only

grouped together under overarching headings (e.g. ‘share information about condition’ and ‘signpost next steps’) that reflect stages of the consultation (as discussed with the CoP). The participant will be asked to rate their perception of the importance of each statement on a 5-point Likert scale.

In Round 2, newly suggested statements in free-text comments, along with emerging findings from iFraP-D (e.g. focus group findings, stakeholder (CoP and PPIE) experiences and views) will be incorporated. Statements identified in Round 1 as being of low importance will be removed. Participants will be shown mean scores of importance from Round 1 and asked to re-rate the importance of each statement, again using the same 5-point numeric Likert scale.

In Round 3, low ranking items will be removed and participants will be asked whether a statement is essential in a time-limited consultation, or not.

Analysis - Patient/carer and clinician responses will be analysed separately. Mean scores will be calculated for Rounds 1 and 2. Items with a mean importance score of less than 4 (range 1-5) in both patient and clinician surveys will be removed after Round 1. Items scoring a mean score of less than 4 in either patient/carer or clinician survey will be individually reviewed by the Study Management Group (including PPIE members) to make a decision about whether the item progresses to Round 2, informed by underpinning theory, other emergent findings in iFraP-D (e.g. focus groups), PPIE member experiences and views, etc.

After Round 2, statements will be ranked according to their mean rating score. The top scoring statements from both patient/carer and clinician surveys will progress to Round 3, identified as those in the top 3 of 4 quartiles. Statements with a patient/carer and/or clinician score in the lowest quartile will be reviewed by the Study Management Group (including PPIE members), as to whether the statement should progress to Round 3, informed by the considerations described in Round 1.

In Round 3, percentage agreement that ‘this item is essential in the time-limited consultation’ will be calculated. Statements will be ranked according to percentage agreement for both patient/carer and clinician surveys. To identify a set number of essential consultation statements, which can realistically be undertaken in a time-limited consultation, we will not pre-define an arbitrary level of agreement for a task to be included in the iFraP consultation. The Study Management Group in conjunction with PPIE members and expert advisors, will review the ranked findings and identify, using their own knowledge and experience and free-text responses, where the most appropriate threshold is for tasks or considerations to be classified as ‘essential’ or ‘optional’.

Step 2: To understand the current clinical context of FLS consultations including barriers and facilitators to change.

This step will involve mixed methods of data collection. A qualitative focus group study and a survey of usual care will achieve objective 3a.

Focus Groups

Objective – To qualitatively explore current practice in relation to fracture prevention communication, anticipated barriers and facilitators to the iFraP intervention and perceived training needs for FLS clinicians

Participants - Two focus groups with patients that have recently attended an FLS consultation and received a treatment recommendation, two focus groups with clinicians that

For peer review only

conduct face-to-face consultations with patients in UK FLSs, and one focus group with GPs with experience of consulting with patients who have been seen in FLS and that work in the catchment of a UK FLS. Focus groups will be conducted with participants from different geographical areas, with approximately 4-8 participants in each group.

Data collection - Focus groups enable investigation of practices among individuals and are particularly suited to studying group norms and processes, as group interaction is explicitly used to generate data and insights.[47] If face-to-face focus groups are inappropriate, qualitative data collection will be conducted remotely (e.g. by telephone or video conferencing software). A topic guide (informed by theory, evidence gathering and CoP/PPIE views) will prompt, facilitate and structure discussion. The guide will include questions related to the TDF as this framework will inform the content of training to enable skills development for clinicians and is therefore relevant to understanding how clinicians would use iFraP. The questions will also be informed by the TFA; our overarching theoretical framework of acceptability enabling the investigation of seven constructs of acceptability. Topic guides will be iteratively developed during the study to account for insights not anticipated.

Analysis - Focus groups will be audio-recorded and transcribed. We will use a framework approach to analysis,[48] including a two-stage process to 1) inductively code transcripts followed by 2) a deductive exercise to map identified codes to the domains of relevant theoretical frameworks, including the TDF, TFA, NPT, in accordance with previous research.[49,50] Theoretical frameworks will provide a lens to deepen our understanding of current FLS practice, intervention acceptability, and barriers to, and facilitators of intervention implementation. For example, TDF domains will be mapped to the COM-B model to identify intervention functions and theory-driven behaviour change techniques.[40] Analysis will be undertaken by two members of the study team independently, with a mapping exercise to explore coding consistency. Findings will be discussed with the wider study team and PAG members. Qualitative data will be managed and coded using NVivo 11.

Usual care survey

Participants - an electronic survey (utilising HealthSurvey, hosted by Keele University) will be designed and distributed to nurses or allied health professionals (AHPs) working in UK FLSs, with a target sample size of 80 across the 4 devolved nations. The survey will be distributed by the Royal College of Physicians Fracture Liaison database national audit (FLS-DB), ROS mailing lists, and by researchers to individuals in their known professional networks.

Data collection - Variation in FLS design and specification influences the amount of clinician-patient dialogue about drug recommendations, which has direct implications for implementation of the iFraP intervention and any subsequent trial. For example, the extent of patient contact (e.g. face-to-face, telephone or by letter) will affect the ability to use the CDST within the consultation. The specific content for the usual care survey will be informed by the focus groups, and will aim to describe and quantify the nature and amount of discussion FLSs have with patients about osteoporotic drugs. Any planned changes in service specification will also be explored.

Analysis - Any duplicate entries from the same FLS will be checked for consistency. If there are any differences the respondent will be emailed to clarify. A descriptive analysis will be undertaken. Findings will be discussed with the CoP to discuss implications for iFraP intervention design.

For peer review only

Results of the descriptive analysis will also be used to create a detailed FLS typology, based on the extent of face-to-face contact to facilitate sampling for the subsequent pilot and feasibility trial.

Step 3: Design and refine the iFraP intervention using field testing

The outputs from studies in Step 1 and 2 will be integrated to inform design of the CDST and training (objective 2, 3b, 3c). iFraP design decision-making will be made and documented by the Study Management Group.[32] Following design of the prototype iFraP consultation intervention (including CDST and training), we will conduct three cycles of in-practice (or field) testing to assess how the fracture prevention components work together, and the acceptability and feasibility of the prototype CDST (objective 3d).

Data integration

Data from the evidence synthesis, Delphi survey and focus groups will be integrated to add rigour, provide greater credibility to results, and generate insights about the intervention in development. Integration will be achieved using a convergent design and merging of the quantitative and qualitative findings using side-by-side joint displays.[51]

A framework for the consultation will be drafted, informed by the consultation stages identified in the evidence synthesis, underpinning theory, frameworks of shared decision-making and principles of health literacy. This will include a series of stages and tasks for the clinician to progress through during the consultation, including gathering information, clinician decision-making, eliciting patient knowledge through to summarising and signposting. Findings from the Delphi analysis (a list of items to be included - and whether they are essential or optional, a list of items excluded, and relevant free-text comments) and qualitative analysis will be mapped to the relevant stage of the consultation in the drafted framework. A separate extraction table will be used for joint display of other contextual considerations for the intervention, derived from the free-text in the Delphi and qualitative findings, and will be organised into considerations with relevance to i) the tool development, ii) the training development and/or iii) subsequent trial design.

The joint display will be interrogated to identify 'meta-inferences'[51]: confirmed, discordant, and expanded findings. Resultant discordant areas, or areas of uncertainty, will be presented to the CoP and PAG for further discussion. Following stakeholder discussion, the list of content and considerations for i) tool development, ii) training development and iii) the trial will be finalised.

iFraP intervention design

Prototype CDST design - The CDST will contain content to support clinician and patient decision-making. The patient-facing component will be designed with the principles of conversation aids, rather than decision aids, to support the discussion between clinician and patient.[52] Frequent stakeholder (CoP and PPIE) workshops throughout iFraP-D will allow members to contribute and provide insight into CDST design. Insights will be considered alongside integration outputs described above in accordance with our informed design approach.[32,39] The prototype tool will be web-based and built using a programmed decision-tree based upon a modified Markov model (a way to model prognosis for clinical problems with ongoing risk). Its development will adhere to international guidance and be informed by a published and process map for DST development.[38] A technical production group will manage the CDST design. A scientific advisory group of osteoporosis academics will advise on the scientific evidence-base that will underpin the CDST. Where possible,

For peer review only

event rates from existing NICE guidelines and meta-analyses will be utilised. PPIE and clinician members of the CoP will informally test early CDST prototypes.

Training design - Separately, also informed by stakeholder (CoP and PPIE) workshops and integration outputs (e.g. list of content and considerations for iFraP development), a training development group (including input from a behavioural psychologist, patients, expert nurse educator and expert in shared decision-making) will map behaviour change techniques targeted to FLS clinician training needs. Qualitative analysis mapped to the TDF [30] aligned with the COM-B model will support identification of theory-driven behaviour change techniques.[29] This process will allow the training development group to develop an evidence-based clinician training package and associated training manual.

In-practice testing

Design - We will conduct three cycles of in-practice testing of the prototype iFraP consultation in one FLS site. Each of the three cycles will consist of five consultations with patients and post-consultation interviews. At the end of each cycle, the FLS clinician conducting the prototype iFraP consultation will also be interviewed. Each consultation will be audio-recorded, observed, and contain a 'think-aloud' interview

Participants - Patients with a recent fragility fracture (n=15) who are referred for an FLS consultation and clinicians conducting face-to-face FLS consultations (up to n=3). In-practice testing will take place at the Haywood Hospital FLS chosen for its geographical proximity to the study team. The Haywood Hospital FLS operates a one-stop model, meaning that if appropriate, patients have a bone density scan (DXA), nurse assessment, drug treatment recommendation, and blood tests as part of one consultation.

Data collection and procedures - Patients with FLS appointments will be asked to consent to having their consultation observed by a researcher and audio-recorded. For each patient participant, the researcher will conduct a brief (previously developed) warm-up exercise on the 'think-aloud' technique prior to the consultation.[53] In each cycle of consultation and 'think aloud' interview, a three step test interview approach will be used.[54] The topic guide will be developed in collaboration with PAG members.

First, the researcher will observe and listen to the test consultation (noting any visual and verbal cues to be explored in the interview immediately following the consultation) and will make notes using an observation schedule. Patients will be asked to mention (aloud, at the time they arise) any thoughts and feelings, if they feel comfortable to do so, during the consultation as part of a 'think-aloud' process.

Second, patient participants will be interviewed on completion of the consultation using pre-defined and spontaneous probes related to iFraP intervention components, to follow-up issues that emerged during the consultation (e.g. to explore unexpected responses and fill in gaps where the participant may not have commented in real-time). This step will provide evidence on whether risk communication is understood by participants in a consistent way and in the way intended, and how well the intervention is meeting its objectives.

Third, participants will be invited to explain comments made in step 2 and their behaviour during the consultation, and share views and opinions of the consultation. They will be encouraged to say everything they thought about each part of the consultation. Participants will be invited to contact the researcher if any further thoughts come to mind after the interview.

For peer review only

Post-consultation interviews with clinicians will be conducted as soon after the last consultation in the cycle as possible. A similar three-step approach will be used. In the first step however, these clinician participants will be played audio clips demonstrating where they explained risk, used the CDST and gave recommendations. They will be encouraged to think-aloud and mention any thoughts or feelings during playback. Second and third steps will be as for patient interviews.

Analysis - The framework approach (as described above for focus groups) will be used. The first interviews will be analysed, with help from CoP and PAG members to interpret data. Relevant changes to iFraP will be made ahead of further testing, supported by theoretical frameworks to identify and overcome barriers to implementation, enabling an iterative cycle moving between user feedback and changes to the intervention.

The outcome of this study will finalise a draft iFraP intervention and intervention manual to be tested in a formal pilot and feasibility trial with nested value of information analysis, to evaluate acceptability, feasibility and cost-effectiveness.

ETHICS AND DISSEMINATION

Ethics approval for the work outlined in this protocol was sought and obtained from North West - Greater Manchester West Research Ethics Committee (reference number: 19/NW/0559).

Dissemination and knowledge mobilisation will be facilitated through national bodies and networks such as the ROS, journal papers and conference presentations. The results of this study will be made widely and freely available to all stakeholders; a summary of the results will be published on the Keele University and ROS website. PAG members will advise on how to translate these into easily understandable messages and on how best to disseminate the results to the wider public.

In addition to publications in open-access peer-reviewed journals, we will use NHS networks and links to professional bodies to support dissemination of the findings to all stakeholders and will use social media to promote the findings via our dedicated Twitter and Facebook feeds.

For peer review only

References

1 National Osteoporosis Society. The Osteoporosis Agenda England: Improving the lives of people with osteoporosis and fragility fractures. 2015.https://theros.org.uk/media/1959/agenda-for-osteoporosis-england-final.pdf

2 NHS RightCare. NHS RightCare scenario: The variation between sub-optimal and optimal pathways. 2017.https://www.england.nhs.uk/rightcare/wp-content/uploads/sites/40/2017/02/rightcare-susans-story-full-narrative.pdf

3 Compston J, Cooper A, Cooper C, *et al.* UK clinical guideline for the prevention and treatment of osteoporosis. *Arch Osteoporos* 2017;**12**:43. doi:10.1007/s11657-017-0324-5

4 National Osteoporosis Society. Effective Secondary Prevention of Fragility Fractures: Clinical Standards for Fracture Liaison Services. 2015.https://theros.org.uk/media/2082/clinical-standards-for-fracture-liaison-services.pdf

5 National Institute for Health and Care Excellence. Bisphosphonates for treating osteoporosis. 2017.https://www.nice.org.uk/guidance/ta464 (accessed 21 Jan 2020).

6 Kanis JA, Svedbom A, Harvey N, *et al.* The Osteoporosis Treatment Gap. *J Bone Miner Res* 2014;**29**:1926–8. doi:10.1002/jbmr.2301

7 Hall SF, Edmonds SW, Lou Y, *et al.* Patient-reported reasons for nonadherence to recommended osteoporosis pharmacotherapy. *J Am Pharm Assoc* 2017;**57**:503–9. doi:10.1016/j.japh.2017.05.003

8 Hiligsmann M, Cornelissen D, Vrijens B, *et al.* Determinants, consequences and potential solutions to poor adherence to anti-osteoporosis treatment: results of an expert group meeting organized by the European Society for Clinical and Economic Aspects of Osteoporosis, Osteoarthritis and Musculoskeletal. *Osteoporos Int* 2019;**30**:2155–65. doi:10.1007/s00198-019-05104-5

9 McHorney CA, Schousboe JT, Cline RR, *et al.* The impact of osteoporosis medication beliefs and side-effect experiences on non-adherence to oral bisphosphonates. *Curr Med Res Opin* 2007;**23**:3137–52.https://www.ncbi.nlm.nih.gov/pubmed/17988435 (accessed 23 Dec 2019).

10 Clark EM, Gould VC, Tobias JH, *et al.* Natural history, reasons for, and impact of low/non-adherence to medications for osteoporosis in a cohort of community-dwelling older women already established on medication: a 2-year follow-up study. *Osteoporos Int* 2016;**27**:579–90. doi:10.1007/s00198-015-3271-2

11 National Osteoporosis Society. Quality Standards for Osteoporosis and Prevention of Fragility Fractures. 2017. https://theros.org.uk/media/0dillsrh/ros-op-standards-november-2017.pdf (accessed 21 Jan 2020).

12 Raybould G, Babatunde O, Evans AL, *et al.* Expressed information needs of patients with osteoporosis and/or fragility fractures: a systematic review. *Arch Osteoporos* 2018;**13**:1–16. doi:10.1007/s11657-018-0470-4

13 Paskins Z, Crawford-Manning F, Cottrell E, *et al.* Acceptability of bisphosphonates among patients, clinicians and managers: A systematic review and framework synthesis. *BMJ Open* 2020;**10**:40634. doi:10.1136/bmjopen-2020-040634

14 Kanis JA, Johnell O, Oden A, *et al.* FRAX^{TM} and the assessment of fracture probability in men and women from the UK. *Osteoporos Int* 2008;**19**:385–97. doi:10.1007/s00198-007-0543-5

For peer review only

15 Salter C, McDaid L, Bhattacharya D, *et al.* Abandoned Acid? Understanding adherence to bisphosphonate medications for the prevention of osteoporosis among older women: A qualitative longitudinal study. *PLoS One* 2014;**9**. doi:10.1371/journal.pone.0083552

16 Gregson CL, Dennison EM, Compston JE, *et al.* Disease-specific perception of fracture risk and incident fracture rates: GLOW cohort study. *Osteoporos Int* 2014;**25**:85–95. doi:10.1007/s00198-013-2438-y

17 Paskins Z, Jinks C, Mahmood W, *et al.* Public priorities for osteoporosis and fracture research: results from a general population survey. *Arch Osteoporos* 2017;**12**:1–8. doi:10.1007/s11657-017-0340-5

18 Street RL, Makoul G, Arora NK, *et al.* How does communication heal? Pathways linking clinician-patient communication to health outcomes. *Patient Educ Couns* 2009;**74**:295–301. doi:10.1016/j.pec.2008.11.015

19 DiMatteo MR, Sherbourne CD, Hays RD, *et al.* Physicians' Characteristics Influence Patients' Adherence to Medical Treatment: Results From the Medical Outcomes Study. *Heal Psychol* 1993;**12**:93–102. doi:10.1037/0278-6133.12.2.93

20 Stacey D, Légaré F, Lewis K, *et al.* Decision aids for people facing health treatment or screening decisions. *Cochrane Database Syst Rev* 2017;**12**:CD001431. doi:10.1002/14651858.CD001431.pub5

21 Elwyn G, Frosch D, Volandes AE, *et al.* Investing in deliberation: A definition and classification of decision support interventions for people facing difficult health decisions. *Med Decis Mak* 2010;**30**:701–11. doi:10.1177/0272989X10386231

22 McCaffery KJ, Holmes-Rovner M, Smith SK, *et al.* Addressing health literacy in patient decision aids. *BMC Med Inform Decis Mak* 2013;**13**:S10. doi:10.1186/1472-6947-13-S2-S10

23 Paskins Z, Torres-Roldan V, Hawarden A, *et al.* Quality and effectiveness of osteoporosis treatment decision aids: a systematic review and environmental scan. *Osteoporos Int* Published Online First: 2020. doi:10.1007/s00198-020-05479-w

24 Kawamoto K, Houlihan CA, Balas EA, *et al.* Improving clinical practice using clinical decision support systems: A systematic review of trials to identify features critical to success. *Br Med J* 2005;**330**:765–8. doi:10.1136/bmj.38398.500764.8f

25 Garg AX, Adhikari NKJ, McDonald H, *et al.* Effects of computerized clinical decision support systems on practitioner performance and patient outcomes: A systematic review. *J Am Med Assoc* 2005;**293**:1223–38. doi:10.1001/jama.293.10.1223

26 Royal Osteoporosis Society. Effective Secondary Prevention of Fragility Fractures: clinical standards for Fracture Liaison Services. 2019.https://theros.org.uk/media/1eubz33w/ros-clinical-standards-for-fracture-liaison-services-august-2019.pdf (accessed 16 Oct 2020).

27 Horne R, Chapman SCE, Parham R, *et al.* Understanding patients' adherence-related Beliefs about Medicines prescribed for long-term conditions: A meta-analytic review of the Necessity-Concerns Framework. *PLoS One* 2013;**8**. doi:10.1371/journal.pone.0080633

28 Sekhon M, Cartwright M, Francis JJ. Acceptability of healthcare interventions: An overview of reviews and development of a theoretical framework. *BMC Health Serv Res* 2017;**17**:1–13. doi:10.1186/s12913-017-2031-8

29 Michie S, van Stralen MM, West R. The behaviour change wheel: A new method for

For peer review only

characterising and designing behaviour change interventions. *Implement Sci* 2011;**6**. doi:10.1186/1748-5908-6-42

30 Cane J, O'Connor D, Michie S. Validation of the theoretical domains framework for use in behaviour change and implementation research. *Implement Sci* 2012;**7**:37. doi:10.1186/1748-5908-7-37

31 Murray E, Treweek S, Pope C, *et al.* Normalisation process theory: A framework for developing, evaluating and implementing complex interventions. *BMC Med* 2010;**8**. doi:10.1186/1741-7015-8-63

32 O'Cathain A, Croot L, Duncan E, *et al.* Guidance on how to develop complex interventions to improve health and healthcare. *BMJ Open* 2019;**9**:e029954. doi:10.1136/bmjopen-2019-029954

33 Campbell NC, Murray E, Darbyshire J, *et al.* Designing and evaluating complex interventions to improve health care. *Br Med J* 2007;**334**:455–9. doi:10.1136/bmj.39108.379965.BE

34 Grol R, Wensing M, Eccles M. *Improving patient care: The implementation of change in clinical practice*. Edinburgh: : Butterworth Heinemann. 2005.

35 O'Cathain A, Croot L, Sworn K, *et al.* Taxonomy of approaches to developing interventions to improve health: a systematic methods overview. *Pilot Feasibility Stud* 2019;**5**:1–27. doi:10.1186/s40814-019-0425-6

36 Croot L, O'Cathain A, Sworn K, *et al.* Developing interventions to improve health: A systematic mapping review of international practice between 2015 and 2016. *Pilot Feasibility Stud* 2019;**5**:1–13. doi:10.1186/s40814-019-0512-8

37 Coulter A, Stilwell D, Kryworuchko J, *et al.* A systematic development process for patient decision aids. *BMC Med Inform Decis Mak* 2013;**13**:S2. doi:10.1186/1472-6947-13-S2-S2

38 Elwyn G, Kreuwel I, Durand MA, *et al.* How to develop web-based decision support interventions for patients: A process map. *Patient Educ Couns* 2011;**82**:260–5. doi:10.1016/j.pec.2010.04.034

39 Rousseau N, Turner KM, Duncan E, *et al.* Attending to design when developing complex health interventions: A qualitative interview study with intervention developers and associated stakeholders. *PLoS One* 2019;**14**:1–20. doi:10.1371/journal.pone.0223615

40 Michie S, Atkins LW. *The Behaviour Change Wheel: A Guide to Interventions*. London: : Silverback Publishing 2014.

41 Wenger E. Communities of Practice: Learning, Meaning, and Identity. *Syst Thinker* 1998;**9**:2–3. doi:10.2277/0521663636

42 Staniszewska S, Brett J, Simera I, *et al.* GRIPP2 reporting checklists: Tools to improve reporting of patient and public involvement in research. *BMJ* 2017;**358**:j3453. doi:10.1136/bmj.j3453

43 Grime JC, Ong BN. Constructing osteoarthritis through discourse - A qualitative analysis of six patient information leaflets on osteoarthritis. *BMC Musculoskelet Disord* 2007;**8**:34. doi:10.1186/1471-2474-8-34

44 Brouwers MC, Kho ME, Browman GP, *et al.* AGREE II: Advancing guideline development, reporting and evaluation in health care. *CMAJ* 2010;**182**:E839. doi:10.1503/cmaj.090449

For peer review only

45 Leventhal H, Benyamini Y, Brownlee S, *et al.* Perceptions of health and illness: Current research and applications. In: Petrie KJ, Weinman J., eds. *Illness representations: Theoretical foundations*. Harwood Academic Publishers. 1997. 19–45.

46 McKenna HP. The Delphi technique: a worthwhile research approach for nursing? *J Adv Nurs* 1994;**19**:1221–5. doi:10.1111/j.1365-2648.1994.tb01207.x

47 Kitzinger J. Qualitative research: introducing focus groups. *BMJ* 1995;**311**:299–302. doi:10.1136/bmj.311.7000.299

48 Ritchie J, Spencer L. Qualitative data analysis for applied policy research. In: Bryman A, Burgess RG, eds. *Analysing qualitative data*. London: : Routledge 1994. 173–94.

49 Saunders B, Hill JC, Foster NE, *et al.* Stratified primary care versus non-stratified care for musculoskeletal pain: Qualitative findings from the STarT MSK feasibility and pilot cluster randomized controlled trial. *BMC Fam Pract* 2020;**21**:1–12. doi:10.1186/s12875-020-1098-1

50 Saunders B, Bartlam B, Foster NE, *et al.* General Practitioners' and patients' perceptions towards stratified care: A theory informed investigation. *BMC Fam Pract* 2016;**17**:125. doi:10.1186/s12875-016-0511-2

51 Younas A, Pedersen M, Durante A. Characteristics of joint displays illustrating data integration in mixed-methods nursing studies. *J Adv Nurs* 2020;**76**:676–86. doi:10.1111/jan.14264

52 Kunneman M, Montori VM, Castaneda-Guarderas A, *et al.* What Is Shared Decision Making? (and What It Is Not). *Acad Emerg Med* 2016;**23**:1320–4. doi:10.1111/acem.13065

53 Beatty P, Willis GB, Beatty PC. Research synthesis: The practice of cognitive interviewing. *Public Opin Q* 2007;**71**:287–311. doi:10.1093/poq/nfm006

54 Paap MCS, Lange L, van der Palen J, *et al.* Using the Three-Step Test Interview to understand how patients perceive the St. George's Respiratory Questionnaire for COPD patients (SGRQ-C). *Qual Life Res* 2016;**25**:1561–70. doi:10.1007/s11136-015-1192-3

For peer review only

Author's contributions

ZP conceived the study. ZP, LB, EC, JF, SL, JJE, EMC, ST, SC, CG, CPI, TON, CM and CJ reviewed the study design and contributed to study implementation as part of the iFraP Study Management Group. JP and RH contributed to study design as expert study advisors. JL, SL, and SR will form the iFraP training development group. ST and SC will inform design and development of the prototype iFraP CDST. CPI will provide health economic expertise. All authors contributed to refinement of the study protocol and approved the final manuscript.

Funding statement and competing interests

This study was funded by the National Institute for Health Research (NIHR) [Clinician Scientist Award (CS-2018-18-ST2-010)/NIHR Academy], the Royal Osteoporosis Society (REF: 430), and the Haywood Rheumatology Research and Development Foundation (not applicable).

Dr Zoe Paskins reports grants from the NIHR Clinician Scientist Award (CS-2018-18-ST2-010), Royal Osteoporosis Society, and the Haywood Rheumatology Research and Development Foundation to conduct this study. Prof Clare Jinks reports funding from the NIHR Applied Research Collaboration (ARC) West Midlands; Prof Christian Mallen reports grants from the NIHR Research Professorship in General Practice (NIHR-RP-2014–04–026), the NIHR School for Primary Care Research and NIHR Applied Research Collaborations (West Midlands), Wellcome, Medical Research Council, Dunhill, Versus Arthritis and Bristol Myer-Squibb, outside the submitted work; Dr Fay Crawford-Manning reports grants from NIHR Clinical Research Network Scholar Programme; Simon Thomas and Prof Stephen Chapman report receiving funds from National Institute for Health Research (NIHR) [Clinician Scientist Award (CS-2018-18-ST2-010)/NIHR Academy] and the Royal Osteoporosis Society via Prescribing Decision Support Ltd to support the development of the iFraP decision support tool; Prof. Terence O'Neill reports grants and non-financial support from AMGEN and the NIHR Manchester Biomedical Research Centre outside the submitted work; Dr Cynthia Iglesias reports grants from NIHR during the conduct of the study and disclosed being a member of NICE's Medical Technologies Advisory Committee outside the submitted work; Dr John Edwards reports grants as a NIHR Academic Clinical Lecturer in Primary Care (CL-2016-10-003). Dr Emma M Clark reports grants from Versus Arthritis, NIHR RfPB, HQIP and the Royal Osteoporosis Society. Dr Laurna Bullock, Dr Elizabeth Cottrell, Prof Robert Horne, Dr Janet Lefroy, Prof Chris Gidlow, Dr Jane Fleming, Prof Sarah Ryan, and Sarah Leyland have nothing to disclose.

The views expressed are those of the author(s) and not necessarily those of the National Health Service, the NIHR, or the Department of Health and Social Care.

Word count: 5617

Exclusive Licence

I, the Submitting Author has the right to grant and does grant on behalf of all authors of the Work (as defined in the below author licence), an exclusive licence and/or a non-exclusive licence for contributions from authors who are: i) UK Crown employees; ii) where BMJ has agreed a CC-BY licence shall apply, and/or iii) in accordance with the terms applicable for US Federal Government officers or employees acting as part of their official duties; on a worldwide, perpetual, irrevocable, royalty-free basis to BMJ Publishing Group Ltd ("BMJ") its licensees and where the relevant Journal is co-owned by BMJ to the co-owners of the Journal, to publish the Work in BMJ Open and any other BMJ products and to exploit all rights, as set out in our licence.

The Submitting Author accepts and understands that any supply made under these terms is made by BMJ to the Submitting Author unless you are acting as an employee on behalf of your employer or a postgraduate student of an affiliated institution which is paying any applicable article publishing charge ("APC") for Open Access articles. Where the Submitting Author wishes to make the Work available on an Open Access basis (and intends to pay the relevant APC), the terms of reuse of such Open Access shall be governed by a Creative Commons licence – details of these licences and which Creative Commons licence will apply to this Work are set out in our licence referred to above.

Figures (uploaded separately)

Fig 1**.** iFraP-D Logic Model

Fig 2**.** Inter-relation between the implementation of change model and iFraP-D data collection methods

For peer review only

Problem: Poor fracture prevention treatment uptake

Targeted determinants: patient health beliefs

Aim: to improve informed decision making in FLS consultations

Intervention Resources

- FLS service
- Evidence summaries
- CDST to depict individualized fracture risk and treatment benefits/harms
- Training package
- Individualised patient information leaflets
- Patient information website

→

Processes

- FLS HCP receives training in using CDST communicating risk, health literacy approaches, eliciting and addressing patient preferences and values and SDM
- Trained HCP conducts FLS consultation using CDST
- Patient given ind. info. leaflet and signposted to website
- GP receives same information

→

Outputs

FLS HCP

- Increased confidence in using CDST, communicating risk, health literacy approaches, eliciting and addressing patient preferences and values and SDM

Post-consultation

- Consistency of messages across primary care and FLS

→

Short term outcomes

CLINICIAN

- Identifies appropriate treatment options

PATIENT

- Increased understanding of own fracture risk & risk/benefit of treatments
- Increased self-efficacy & enablement
- Trust in clinician

↓

- Reduced decisional conflict
- Improved decision quality
- Increased rates of treatment initiation

→

Long term outcomes

PATIENT

- Persistence with fracture prevention treatments at 12 months
- Lifestyle outcomes (PA, smoking, alcohol)
- *Increased incidence of drug adverse events*
- Fracture reduction

NHS

- Guideline adherence
- Reduced costs of fracture care

Context: Consultations conducted in pre-existing specialist face-to-face fracture prevention services (Fracture Liaison Service - FLS)
Contextual factors: Poor uptake of NICE/NOGG guidelines; disconnect in advice given to patient between FLS and primary care; media and wider social influences on health behaviours

**CDST computerised decision support tool, FLS Fracture Liaison Service, HCP healthcare professional, PA physical activity, GP general practitioner, SDM shared decision-making*

For peer review only - http://bmjopen.bmj.com/site/about/guidelines.xhtml

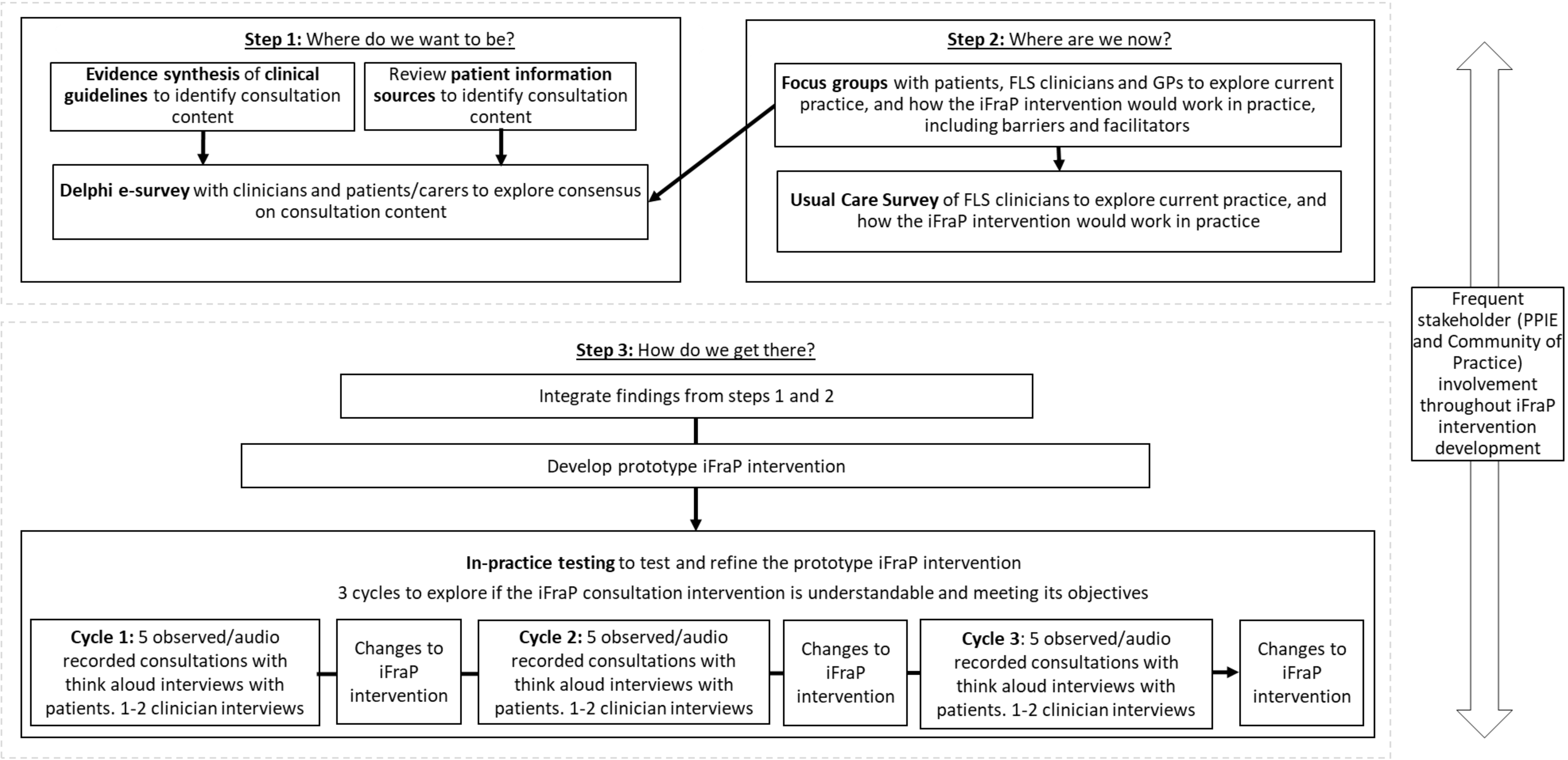
Step 1: Where do we want to be?
Evidence synthesis of clinical guidelines to identify consultation content
Review patient information sources to identify consultation content
Delphi e-survey with clinicians and patients/carers to explore consensus on consultation content
Step 2: Where are we now?
Focus groups with patients, FLS clinicians and GPs to explore current practice, and how the iFraP intervention would work in practice, including barriers and facilitators
Usual Care Survey of FLS clinicians to explore current practice, and how the iFraP intervention would work in practice
Step 3: How do we get there?
Integrate findings from steps 1 and 2
Develop prototype iFraP intervention
In-practice testing to test and refine the prototype iFraP intervention
3 cycles to explore if the iFraP consultation intervention is understandable and meeting its objectives
Cycle 1: 5 observed/audio recorded consultations with think aloud interviews with patients. 1-2 clinician interviews
Changes to iFraP intervention
Cycle 2: 5 observed/audio recorded consultations with think aloud interviews with patients. 1-2 clinician interviews
Changes to iFraP intervention
Cycle 3: 5 observed/audio recorded consultations with think aloud interviews with patients. 1-2 clinician interviews
Changes to iFraP intervention
Frequent stakeholder (PPIE and Community of Practice) involvement throughout iFraP intervention development

Supplementary Table 1. Eligibility criteria for osteoporosis guideline evidence synthesis

	**Inclusion criteria**
**Population**	Patients at risk of osteoporosis or with fragility fractures
**Interventions**	Clinician-patient consultations to assess risk and initiate/recommend treatment for fracture prevention
**Outcomes**	Assessment<br>Explanations<br>Decision-making
**Setting**	Relevant to UK primary or secondary care (e.g. UK, European or international)
**Date**	Those guidelines that have been developed, reviewed or revised within the past 10 years will be used (2009 to Feb 2019)
	**Exclusion criteria**
Guidelines relating to:<br>• managing patients already on fracture prevention treatments<br>• disease specific conditions (e.g. screening for osteoporosis in inflammatory bowel disease)<br>• patients with one specific fracture site (e.g. hip fracture)<br>• steroid-induced osteoporosis<br>• specific countries other than UK<br>• fracture assessment and management<br>Guidelines related to pre-clinical and animal studies<br>Withdrawn guidance<br>The original research manuscripts underpinning the guidelines<br>Studies not in the English language.	

For peer review only

BMJ Open

Improving uptake of Fracture Prevention drug treatments: a protocol for Development of a consultation intervention (iFraP-D)

Journal:	*BMJ Open*
Manuscript ID	bmjopen-2021-048811.R1
Article Type:	Protocol
Date Submitted by the Author:	26-Jul-2021
Complete List of Authors:	Paskins, Zoe; Keele University, School of Medicine; Haywood Hospital, Academic Rheumatology Centre<br>Bullock, Laurna; Keele University, School of Medicine<br>Crawford-Manning, Fay; Keele University, School of Medicine; Haywood Hospital, Academic Rheumatology Centre<br>Cottrell, Elizabeth ; Keele University, School of Medicine<br>Fleming, Jane; University of Cambridge, Cambridge Public Health; Addenbrooke's Hospital, Cambridge University Hospitals NHS Trust<br>Leyland, Sarah; Royal Osteoporosis Society<br>Edwards, John; Keele University, School of Medicine<br>Clark, Emma; University of Bristol, Bristol Medical School<br>Thomas, Simon; Keele University, School of Pharmacy and Bioengineering<br>Chapman, Stephen; Keele University, School of Pharmacy and Bioengineering<br>Ryan, Sarah; Keele University, School of Medicine & School of Nursing and Midwifery; Haywood Hospital, Academic Rheumatology Centre<br>Lefroy, JE; Keele University, School of Medicine<br>Gidlow, Christopher; Staffordshire University, Centre for Health and Development<br>Iglesias, C; University of York, Department of Health Sciences; Aalborg Universitet, Danish Centre for Healthcare Improvements<br>Protheroe, Joanne; Keele University, School of Medicine<br>Horne, Robert; University College London, Centre for Behavioural Medicine, UCL School of Pharmacy<br>O'Neill, Terence; University of Manchester, Centre for Epidemiology Versus Arthritis; Manchester University NHS Foundation Trust, NIHR Manchester Biomedical Research Centre<br>Mallen, Christian; Keele University, School of Medicine<br>Jinks, Clare; Keele University, School of Medicine
**Primary Subject Heading**:	Rheumatology
Secondary Subject Heading:	Communication, Health services research, Patient-centred medicine, Qualitative research
Keywords:	RHEUMATOLOGY, Musculoskeletal disorders < ORTHOPAEDIC & TRAUMA SURGERY, QUALITATIVE RESEARCH

SCHOLARONE™
Manuscripts

I, the Submitting Author has the right to grant and does grant on behalf of all authors of the Work (as defined in the below author licence), an exclusive licence and/or a non-exclusive licence for contributions from authors who are: i) UK Crown employees; ii) where BMJ has agreed a CC-BY licence shall apply, and/or iii) in accordance with the terms applicable for US Federal Government officers or employees acting as part of their official duties; on a worldwide, perpetual, irrevocable, royalty-free basis to BMJ Publishing Group Ltd ("BMJ") its licensees and where the relevant Journal is co-owned by BMJ to the co-owners of the Journal, to publish the Work in this journal and any other BMJ products and to exploit all rights, as set out in our licence.

The Submitting Author accepts and understands that any supply made under these terms is made by BMJ to the Submitting Author unless you are acting as an employee on behalf of your employer or a postgraduate student of an affiliated institution which is paying any applicable article publishing charge ("APC") for Open Access articles. Where the Submitting Author wishes to make the Work available on an Open Access basis (and intends to pay the relevant APC), the terms of reuse of such Open Access shall be governed by a Creative Commons licence – details of these licences and which Creative Commons licence will apply to this Work are set out in our licence referred to above.

Other than as permitted in any relevant BMJ Author's Self Archiving Policies, I confirm this Work has not been accepted for publication elsewhere, is not being considered for publication elsewhere and does not duplicate material already published. I confirm all authors consent to publication of this Work and authorise the granting of this licence.

For peer review only

Title

Improving uptake of Fracture Prevention drug treatments: a protocol for Development of a consultation intervention (iFraP-D)

Authors

Dr Zoe Paskins. School of Medicine, Keele University & Haywood Academic Rheumatology Centre, Staffordshire and Stoke-on-Trent Partnership Trust, Stoke-on-Trent, Staffordshire, UK. ORCID: 0000-0002-7783-2986

Dr Laurna Bullock. School of Medicine, Keele University, Staffordshire, UK. ORCID: 0000-0002-4193-1835

Dr Fay Crawford-Manning. School of Medicine, Keele University & Haywood Academic Rheumatology Centre, Staffordshire and Stoke-on-Trent Partnership Trust, Stoke-on-Trent, Staffordshire, UK. ORCID: 0000-0002-9768-1695

Dr Elizabeth Cottrell. School of Medicine, Keele University, Staffordshire, UK. ORCID: 0000-0002-5757-1854

Dr Jane Fleming. Cambridge Public Health, University of Cambridge & Addenbrooke's Hospital Fracture Liaison Service, Cambridge University Hospitals NHS Trust, Cambridge, UK. ORCID: 0000-0002-8127-2061

Sarah Leyland. Royal Osteoporosis Society, Bath, UK

Dr John J Edwards. School of Medicine, Keele University, Staffordshire, UK. ORCID: 0000-0003-0432-7783

Dr Emma M Clark. Bristol Medical School, Faculty of Health Sciences, University of Bristol, Bristol, UK. ORCID 0000-0001-8332-9052

Simon Thomas. School of Pharmacy and Bioengineering, Keele University, Staffordshire, UK

Prof Stephen Chapman. School of Pharmacy and Bioengineering, Keele University, Staffordshire, UK

Prof Sarah Ryan. School of Medicine & School of Nursing and Midwifery, Keele University & Haywood Academic Rheumatology Centre, Staffordshire and Stoke-on-Trent Partnership Trust, Stoke-on-Trent, Staffordshire, UK

Prof Janet Lefroy. School of Medicine, Keele University, Staffordshire, UK. ORCID: 0000-0002-2662-1919

Prof Christopher Gidlow. Centre for Health and Development, Staffordshire University, Stoke-on-Trent, Staffordshire, UK. ORCID: 0000-0003-4990-4572

Dr Cynthia P Iglesias. Department of Health Sciences, University of York, York, UK & Danish Centre for Healthcare Improvements (CHI), Aalborg University, Denmark. ORCID: 0000-0002-3426-0930

Prof Joanne Protheroe. School of Medicine, Keele University, Staffordshire, UK. ORCID: 0000-0002-9608-1487

Prof Robert Horne. Centre for Behavioural Medicine, UCL School of Pharmacy, University College London, London, UK. ORCID: 0000-0002-3068-8438

For peer review only - http://bmjopen.bmj.com/site/about/guidelines.xhtml

Prof Terence W O'Neill. Centre for Epidemiology Versus Arthritis, University of Manchester & NIHR Manchester Biomedical Research Centre, Manchester University NHS Foundation Trust, Manchester, UK. ORCID: 0000-0002-8896-4677

Prof Christian Mallen, School of Medicine, Keele University, Staffordshire, UK. ORCID: 0000-0002-2677-1028

Prof Clare Jinks, School of Medicine, Keele University, Staffordshire, UK. ORCID: 0000-0002-3407-2446

Correspondence:

Zoe Paskins z.paskins@keele.ac.uk

Tel 000 44 1782 733975 Fax: 00 44 1782 734719

Word count: 5649

For peer review only

Abstract

Introduction

Prevention of fragility fractures, a source of significant economic and personal burden, is hindered by poor uptake of fracture prevention medicines. Enhancing communication of scientific evidence and elicitation of patient medication related beliefs has the potential to increase patient commitment to treatment. The iFraP (**I**mproving uptake of **Fra**cture **P**revention drug treatments) programme aims to develop and evaluate a theoretically informed, complex intervention consisting of a computerised web-based decision support tool, training package and information resources, to facilitate informed decision-making about fracture prevention treatment, with a long-term aim of improving informed treatment adherence. This protocol focuses on the iFraP **D**evelopment (iFraP-D) work.

Methods and analysis

The approach to iFraP development is informed by the Medical Research Council complex intervention development and evaluation framework and the three-step implementation of change model. The context for the study is UK Fracture Liaison Services (FLS), which enact secondary fracture prevention. An evidence synthesis of clinical guidelines and Delphi exercise will be conducted to identify content for the intervention. Focus groups with patients, FLS clinicians and General Practitioners and a usual care survey will facilitate understanding of current practice, and investigate barriers and facilitators to change. Design of the iFraP intervention will be informed by decision aid development standards and theories of implementation, behaviour change, acceptability and medicines adherence. The principles of co-design will underpin all elements of the study through a dedicated iFraP Community of Practice including key stakeholders and Patient Advisory Group. In-practice testing of the prototype intervention will inform revisions ready for further testing in a subsequent pilot and feasibility randomised trial.

Ethics and dissemination

Ethical approval was obtained from North West - Greater Manchester West Research Ethics Committee (19/NW/0559). Dissemination and knowledge mobilisation will be facilitated through national bodies and networks, publications and presentations.

Registration

Research Registry (identifying number: researchregistry5041)
https://www.researchregistry.com/browse-the-registry#home/registrationdetails/5d3af8eb65383300105d9e68/

Article Summary

Strengths and limitations of this study

- A robust intervention development process will incorporate multiple sources of evidence, informed by the Medical Research Council framework for developing complex interventions and a three step model of change.
- A comprehensive logic model and use of the Necessity Concerns Framework, provides a theoretical basis for enhancing informed adherence.

For peer review only - http://bmjopen.bmj.com/site/about/guidelines.xhtml

- Collaboration with patients and clinicians, using the principles of co-design, and use of the Theoretical Framework of Acceptability to analyse qualitative data will enable us to iteratively develop an intervention that is relevant and acceptable to users.
- iFraP will be designed to address barriers to implementation from the outset, through the use of Normalisation Process Theory, Theoretical Domains Framework, and COM-B, and will produce evidence on barriers and facilitators to implementation.
- The research will be conducted in the UK, with in-practice testing conducted at one site; it is possible that barriers and facilitators to change, and the relevance of our intervention may vary across FLS sites, different contexts (e.g. in primary care) and geographical locations (nationally and internationally).

For peer review only

For peer review only - http://bmjopen.bmj.com/site/about/guidelines.xhtml

INTRODUCTION

In the UK, three million people are estimated to have osteoporosis,[1] contributing to over 500,000 fragility fractures (fractures resulting from low trauma) per year, costing an estimated £4.4 billion per annum.[2] Fragility fractures can be devastating, sometimes resulting in loss of independence and mortality.[3] Hip fractures alone account for 85,000 unplanned hospital admissions and 1.8 million bed-days in the UK per year.[4] Evidence-based treatments, such as bisphosphonates, are recommended by the National Institute for Health and Care Excellence (NICE) for patients with osteoporosis and/or a high fracture risk. They are inexpensive, cost-effective, readily available and reduce fracture risk by 20-70% (depending on fracture site).[5] Despite this, a treatment gap exists. Up to 80% of patients who experience a fragility fracture do not receive medication in the year following fracture,[6] 25% of people who are offered medication decline it (non-initiation),[7] and among those who do start bisphosphonates, few persist for long enough for it to be effective, with adherence estimated at 16-60% at one year.[8] Closing this treatment gap may prevent at least 20,000 hip fractures annually in the UK.[4]

Patient reasons for non-initiation and non-persistence of oral bisphosphonates (the mainstay of osteoporosis treatment) are complex and include: perceptions that drugs are not effective, not necessary and/or not safe; limited understanding of the consequences of non-treatment; and, concerns about perceived or experienced side effects.[9,10] Despite national osteoporosis guidance recommending the provision of information as a core component of management,[11] patients report that osteoporosis information provided in consultations is often not easy to understand.[12] Some primary care clinicians believe that bisphosphonates are not safe, effective or necessary.[13] The more recent shift to base treatment recommendations on fracture risk rather than bone density readings,[14] is not without challenge: patients struggle to understand fracture risk assessments[15] and frequently underestimate their risk of fracture.[16] This suggests unmet health literacy needs and patients have identified improving access to information from health professionals as the number one patient priority for osteoporosis research.[17]

Patients ultimately decide whether to start and continue taking medication, but this decision-making is influenced by the clinician-patient interaction. In order to decide to start and persist with medication, patients need to believe that recommended drug treatment is necessary, relevant, safe and practicable. Effective communication that enables patients to understand complex medical terms and concepts in lay terms, increases patient satisfaction, facilitates participation in the consultation, promotes trust[18] and may increase patients' commitment to medication.[19] This highlights the relevance of promoting and supporting effective communication between clinician and patients, and suggests that improving communication of the harms and benefits of osteoporosis medications may be beneficial in reducing the treatment gap.

Decision aids (DAs) include numerical estimates of risk/benefit. They can facilitate improved risk communication and support patient decision-making before or during health care consultations.[20,21] When used across a range of conditions, DAs can: increase patient knowledge, reduce decisional conflict, increase patient participation in decision-making and improve the accuracy of risk perception.[20] A recent Cochrane review reported high-quality evidence that DAs, across a range of conditions, increase patient knowledge and reduce decisional conflict, and moderate-quality evidence that DAs increase patient participation in decision-making and improve the accuracy of risk perception.[20] Increases in knowledge and informed choice were also reported in studies where health literacy needs were addressed.[22] Evidence from pooled analyses of studies where there was no equipoise

For peer review only - http://bmjopen.bmj.com/site/about/guidelines.xhtml

(meaning that DAs were used to give information about recommended drug treatments, rather than to choose between treatment options with perceived equal benefits), has indicated that DAs improve treatment initiation rates.[20] In osteoporosis, use of DAs can increase accuracy of risk perception and shared decision-making. However, existing osteoporosis DAs fail to comprehensively meet international quality standards and patient needs,[23] underlining the requirement for further development.

DAs are sometimes called Decision Support Tools (DSTs), particularly when tools also contain clinician decision support. DSTs can also support clinicians to follow evidence-based practice and have been shown to improve adherence to evidence-based guidelines.[24,25]

The iFraP (**I**mproving uptake of **Fra**cture **P**revention drug treatments) programme, aims to develop and evaluate a theoretically informed, complex intervention consisting of a computerised (C)DST, training package and information resources, to facilitate informed decision-making about fracture prevention treatment, with a long-term aim of improving informed treatment adherence. This protocol is for the first study within the iFraP programme of work focusing on the **D**evelopment of the new consultation intervention, referred to as iFraP-**D**.

For peer review only - http://bmjopen.bmj.com/site/about/guidelines.xhtml

STUDY PROTOCOL

Aims and Objectives

The iFraP intervention development study (iFraP-D) has three overarching objectives, which are to:

1. Develop core content for a new consultation intervention (iFraP) based on theory, published guidance, systematic review evidence, a Delphi consensus exercise and stakeholder engagement
2. Design a prototype CDST using published guidance and with stakeholder engagement
3. Use qualitative methods and stakeholder engagement to:
    a. Investigate current practice, and the barriers and facilitators to, communicating fracture risk and treatment benefits/harms and to delivering the iFraP intervention (CDST and training package), including the training needs of clinicians
    b. Co-design the components of the iFraP intervention and associated training package for clinicians
    c. Plan the integration of components in the new iFraP consultation intervention
    d. Conduct cycles of in-practice testing to determine if the iFraP intervention is meeting its objectives, with subsequent refinement of the intervention.

Overview of context and intervention

The proposed iFraP consultation intervention will be primarily designed for Fracture Liaison Services (FLSs). These are usually nurse-led, and address secondary fracture prevention by: systematically identifying patients with fragility fractures; assessing the patient's bone health, risk of falls and future fracture; and providing treatment recommendations to the patient and primary care. Clinical standards for FLS also recommend follow-up consultations, three and 12 months post-fracture, to support medicines adherence.[26] Within the context of FLS, all consultations are concerned with future fracture prevention, which will maximise efficiency of recruitment and testing. The multidisciplinary study team will allow the relevance of the intervention for use in other primary and secondary care settings in which fracture prevention treatments are recommended to patients, to be considered.

The iFraP consultation intervention will support clinicians to:

- Make decisions about who is eligible for fracture prevention treatment, using a CDST to operationalise existing clinical guidelines
- In patients in whom fracture prevention treatment is indicated:
    - Communicate the risks and benefits of fracture prevention treatment, including individualised fracture risk, using a CDST, adopting universal precautions for low health literacy
    - Elicit patients' understanding, and concerns about fracture prevention treatment
    - Assess readiness to initiate fracture prevention treatments and facilitate behaviour change.

For peer review only - http://bmjopen.bmj.com/site/about/guidelines.xhtml

- Communicate consultation outcomes with the patients' primary care provider and facilitate consistent information provision across primary and secondary care

The iFraP consultation intervention will be delivered within FLS consultations and will include:

- A CDST to communicate individual fracture risk. This will include clinician decision-support and a patient-facing DA. It will be dynamic, interactive and tailored to risks and needs of the patient. It will incorporate fracture risk (calculated in external systems (e.g. FRAX)), an indicator for clinicians of whether treatment is recommended, a pictorial presentation of individualised fracture risk, fracture risk with medication (to show benefits of treatment), and possible treatment harms. The CDST will be used by trained clinicians in a model (face-to-face or remote) consultation with patients.
- Clinician training in delivering the consultation intervention, and supporting delivery of follow-up consultations. This will encompass a prioritised list of key tasks for the clinician (both information giving and eliciting) to undertake. The training will include face-to-face sessions and an e-learning package to introduce the intervention, coach clinicians in listening skills, shared decision-making skills and universal precautions for health literacy, and provide opportunities to practice using the CDST.
- Information resources (paper and online) for the patient and GP to refer to after the initial or follow-up consultation, including a printout from the CDST.

Theoretical frameworks underpinning iFraP development

The long-term aim of the iFraP intervention is to improve fracture prevention treatment uptake, thereby preventing future fragility fractures. The intervention is targeted for use by clinicians together with the patient. This will empower clinicians to support patient behaviour change, and support clinicians with the skills to elicit and address patient perceptions and practicalities related to treatment adherence. The Necessity-Concerns Framework (NCF) will be used as an overarching theoretical framework when designing the iFraP intervention to understand patient's attitudes and beliefs underpinning treatment non-adherence.[27] The NCF suggests that medication adherence is influenced by implicit judgements of personal need for the treatment (necessity beliefs) and concerns about the potential consequences of treatment.[27]

To optimise iFraP in terms of acceptability to clinicians and patients, we will use the Theoretical Framework of Acceptability (TFA) [28] as an overarching framework to inform iFraP intervention design. The TFA consists of seven constructs of acceptability of healthcare interventions (affective attitude, burden, intervention coherence, ethicality, perceived expectations, opportunity cost, and self-efficacy). These constructs will inform data collection and analysis.

The implementation of iFraP is informed by the COM-B model of behaviour change [29] and the complementary Theoretical Domains Framework (TDF)[30] which aim to simplify and integrate a range of behaviour change theories. The TDF identifies 14 related domains of influence on behaviour. In iFraP-D, data collection will be informed by domains within the TDF (e.g. knowledge, beliefs about capabilities, skills, and goals). This will identify potential barriers and enablers for clinicians to implement iFraP ahead of a formal feasibility study. Normalisation Process Theory (NPT)[31] will underpin the investigation of the dynamics of implementing, embedding, and integrating iFraP, in order to identify potential process problems related to implementing iFraP in the next phase of feasibility testing. NPT ensures consideration of potential structural problems about the integration of iFraP into existing services.

For peer review only - http://bmjopen.bmj.com/site/about/guidelines.xhtml

The specific programme theory for the iFraP-D study is detailed in the study logic model (Figure 1). This initial logic model may undergo iterative changes throughout the iFraP programme. Throughout development, a health economist will define the decision problem enabling identification of the different elements of treatment effect potentially associated with the iFraP intervention and directly informing the data collection strategy, including primary and secondary outcomes, for the subsequent pilot and feasibility trial that will follow iFraP-D.

Approach to iFraP intervention development

In line with key principles of intervention development, our approach will be iterative, open to change and forward looking to future evaluation and implementation.[32] The Medical Research Council guidance on complex intervention development will be used as an overall framework,[33] within which we will specifically use a three-step implementation of change model.[34] This approach incorporates three pragmatic questions with three steps, each linked to iFraP study objectives outlined previously:

- **Step 1**: 'Where do we want to be?' Make a concrete proposal for change, and develop the content and format of the consultation intervention (objective 1).
- **Step 2:** 'Where are we now?' - Understand the current clinical context including barriers and facilitators to change (objective 3a).
  Step 3: 'How do we get there' – Develop a strategy to change behaviours, by designing and refining the prototype into a draft intervention, and field-testing (objective 2, 3b, 3c and 3d).

To answer the above questions we will take actions common to different intervention development approaches;[35,36] drawing upon theory, empirical evidence from the evidence synthesis, Delphi survey, qualitative focus groups and in-practice testing, stakeholder engagement, and guidance for the development of DAs.[37,38] We will adopt an informed design to iFraP design decision-making.[32,39] We will frequently engage with stakeholders as part of our Community of Practice (CoP) and Patient Advisory Groups (PAG) to discuss ideas generated by the study team and research findings.[39] The informed design will facilitate stakeholder involvement in iFraP intervention content and design decisions,[39] with final design decision-making made and documented by the Study Management Group [32] supported by the APEASE framework where appropriate.[40]

Each step and linked study objective are outlined in turn throughout this protocol. The inter-relation between the three steps and methods of data collection is displayed in Figure 2.

Co-design of the iFraP intervention

Community of Practice (CoP)

CoPs bring together expertise with a common concern or interest, with the aim of improving and learning to do better through regular group interaction.[41] We will bring together relevant expertise (FLS clinicians, GPs, osteoporosis specialists, commissioning representatives, patients (with experience of fracture prevention treatment, supported by a PPIE worker)), representatives from the Royal Osteoporosis Society (ROS) and Health Literacy UK and a behaviour change expert) in a stakeholder group that adopts a CoP approach.[41] The CoP will provide their views on the iFraP intervention content, and discuss the findings from the evidence synthesis, Delphi study, qualitative focus groups and in-practice testing.

Patient and Public Involvement and Engagement (PPIE)

For peer review only

For peer review only - http://bmjopen.bmj.com/site/about/guidelines.xhtml

The osteoporosis Research User Group (RUG) at Keele University comprises patients with experience of osteoporosis and/or fragility fractures, or carers for such patients. These RUG members had substantial involvement in a previous study to identify patient and public priorities for research in osteoporosis, which provided the starting point for iFraP.[17] Furthermore, the study specific PAG informed and agreed how PPIE members will be involved throughout the iFraP programme at the outset. In addition to PAG members participating in the Study Management Group, Award Steering Committee meetings, and CoP meetings, approximately six study specific PAG meetings will be convened throughout the iFraP research cycle. PAG meetings will: i) facilitate design, analysis and interpretation of the Delphi study (including how best to recruit and explain the Delphi study to patients, piloting the survey, and contributing to analysis), qualitative focus groups and in-practice testing (by informing recruitment, co-designing topic guide content and supporting analysis and interpretation of findings); ii) co-design visual components of the CDST; iii) informally test the CDST prototype before in-practice testing; and, iv) provide advice about dissemination to the wider public, including what results to share, when, and in what format. Additional information regarding PPIE is detailed throughout this protocol where appropriate. The GRIPP2 guidelines will be used in future dissemination to guide PPIE reporting.[42]

METHODS AND ANALYSIS

The iFraP-D study began at the end of March 2019 and the study is expected to be completed in December 2021.

Step 1: Developing content and format of iFraP

In order to inform the content of the model consultation and the CDST components (overarching objective 1) of the iFraP consultation intervention, an evidence synthesis will be conducted of clinical guidelines, followed by consultation with stakeholders and a Delphi study with patients/carers and clinicians.

Evidence synthesis and stakeholder consultation

Search strategy - To identify osteoporosis clinical guidelines, the NHS Evidence electronic database will be searched using keywords ‘fragility fracture’ and ‘osteoporosis’ and ‘guidelines’ for studies that fulfilled the eligibility criteria (Supplementary Table 1). The search will be filtered to include guidance, quality indicators, and policy and strategy. Guidelines that were developed, reviewed or revised within the 10 years prior to search inception will be used. Generic NICE guidance relating to conducting the consultation will also be included.

Patient information resources will be identified using a Google search. Leaflets and webpages will be selected because they are easily available (e.g. on the internet, through patient organisations or places that people with osteoporosis might visit such as pharmacies). They will come from three different types of source: health care providers (UK National Health Service), charitable and voluntary organisations, and the medical profession.

Selection process - Eligible guidelines will be selected on title first by one reviewer (ZP). Full texts will be retrieved and assessed if the title, summary or abstract provides insufficient information.

Eligible patient information resources will be selected by two reviewers. A pragmatic and purposive sample of resources that are most commonly accessed and represent the three groups of information providers will be selected.[43]

Quality appraisal - The quality of the included guidelines will be appraised using the AGREE II tool.[44] Guidelines that score 75% or above will be tagged as high quality (in line with

For peer review only - http://bmjopen.bmj.com/site/about/guidelines.xhtml

examples given by the AGREE II developers); this quality score will inform discussion in the stakeholder groups about the relevance of recommendations.

The overall quality of each patient information resource will be assessed using a modified International Patient Decision Aid Standard (m-IPDAS).[37] The m-IPDAS assess patient information based on 32 items across 8 categories of: content, bias, detail, probabilities, accuracy, decision-making, conflict of interest, structure and layout, and reliability.

Data extraction - Statements and recommendations from included guidelines that are relevant to tasks for the clinician in the consultation will be extracted and grouped into steps of phases of the consultation. Text from patient information resources will be extracted relating to descriptions of osteoporosis and osteoporosis drug treatment using a bespoke data extraction form in excel.

Analysis - A narrative summary will report the findings of the guideline evidence synthesis, including textual description of guidelines, tabulation of recommendations and exploration of relationships between and within guidelines.

Extracted data from the patient information will be thematically analysed independently by two members of the study team using the five domains of Leventhal's Common-Sense Model of Disease as a deductive framework.[45] Discussions will check the consistency of coding against the framework.

Stakeholder consultation - Evidence synthesis findings will be presented to the CoP for reflection on the findings and discussion of any inconsistencies, and discussion of how clinician tasks map to stages of the FLS consultation. The synthesis output (informed by the CoP) will form the basis of the Delphi.

Delphi Study

Design - Three rounds of a modified Delphi consensus study [46] will be conducted. The Delphi approach is described as 'modified', as participants will be presented with a list of statements to consider rather than generating their own list. However, in the first round, they will also have the opportunity to suggest additional statements for the consultation in free-text boxes. The aim of the Delphi is not only to make decisions about inclusion and exclusion but also, for included statements, to ascertain essential and optional consultation content. The same survey will be developed for all participants (both clinical and patient/carer) to develop a common, understandable language. Survey participants will be presented with clinical vignettes and a list of potential content for the model consultation. The content will be derived from the evidence synthesis, informed by the CoP, and written in partnership with PAG members.

Participants - Patients with osteoporosis and/or fragility fractures and/or their carers will be recruited through the ROS supporters' network. Clinicians who have experience of consulting with patients, where fracture risk is calculated and fracture prevention drug treatments are recommended, will be invited to participate through ROS clinician mailing lists and the study team's clinical networks. Clinicians will be multidisciplinary; from primary and secondary care and academic settings. We aim to invite up to 400 participants, anticipating a 40% initial response rate, and subsequent drop out at Rounds 2 and 3, with the aim of achieving a final sample of 15 patients/carers and 15 clinicians.

Data collection and analysis - In Round 1, statements will be presented that relate to tasks of the consultation, to include clinician decision-making tasks and considerations, eliciting information, giving information, example explanations and hypothetical use of the CDST, for

For peer review only - http://bmjopen.bmj.com/site/about/guidelines.xhtml

example, ‘the patient should be verbally informed of their individualised fracture risk’, and ‘the patient should be shown a picture of their individualised fracture risk’. Statements will be grouped together under overarching headings (e.g. ‘share information about condition’ and ‘signpost next steps’) that reflect stages of the consultation (as discussed with the CoP). The participant will be asked to rate their perception of the importance of each statement on a 5-point Likert scale.

Patient/carer and clinician responses will be analysed separately. Mean scores will be calculated. Items with a mean importance score of less than 4 (range 1-5) in both patient and clinician surveys will be removed. Items scoring a mean score of less than 4 in either patient/carer or clinician survey will be individually reviewed by the Study Management Group (including PPIE members) to make a decision about whether the item progresses to Round 2, informed by underpinning theory, other emergent findings in iFraP-D (e.g. focus groups), PPIE member experiences and views, etc.

In Round 2, newly suggested statements in free-text comments, along with emerging findings from iFraP-D (e.g. focus group findings, stakeholder (CoP and PPIE) experiences and views) will be added to statements progressing from Round 1. Participants will be shown mean scores of importance from Round 1 and asked to re-rate the importance of each statement, again using the same 5-point numeric Likert scale.

After Round 2, statements will be ranked according to their mean rating score (calculated as per Round 1). The top scoring statements from both patient/carer and clinician surveys will progress to Round 3, identified as those in the top 3 of 4 quartiles. Statements with a patient/carer and/or clinician score in the lowest quartile will be reviewed by the Study Management Group (including PPIE members), as to whether the statement should progress to Round 3, informed by the considerations described in Round 1.

In Round 3, low ranking items will be removed and participants will be asked to agree/disagree whether a statement is essential in a time-limited consultation, or not. Percentage agreement that ‘this item is essential in the time-limited consultation’ will be calculated. Statements will be ranked according to percentage agreement for both patient/carer and clinician surveys. To identify a set number of essential consultation statements, which can realistically be undertaken in a time-limited consultation, we will not pre-define an arbitrary level of agreement for a task to be included in the iFraP consultation. The Study Management Group in conjunction with PPIE members and expert advisors, will review the ranked findings and identify, using their own knowledge and experience and free-text responses, where the most appropriate threshold is for tasks or considerations to be classified as ‘essential’ or ‘optional’.

Step 2: To understand the current clinical context of FLS consultations including barriers and facilitators to change.
This step will involve mixed methods of data collection. A qualitative focus group study and a survey of usual care will achieve objective 3a.

Focus Groups

Objective – To qualitatively explore current practice in relation to fracture prevention communication, anticipated barriers and facilitators to the iFraP intervention and perceived training needs for FLS clinicians

Participants - Two focus groups with patients that have recently attended an FLS consultation and received a treatment recommendation, two focus groups with clinicians that conduct face-to-face consultations with patients in UK FLSs, and one focus group with GPs

For peer review only - http://bmjopen.bmj.com/site/about/guidelines.xhtml

with experience of consulting with patients who have been seen in FLS and that work in the catchment of a UK FLS. Focus groups will be conducted with participants from different geographical areas, with approximately 4-8 participants in each group.

Data collection - Focus groups enable investigation of practices among individuals and are particularly suited to studying group norms and processes, as group interaction is explicitly used to generate data and insights.[47] If face-to-face focus groups are inappropriate, qualitative data collection will be conducted remotely (e.g. by telephone or video conferencing software) and audio-recorded. A topic guide (informed by theory, evidence gathering and CoP/PPIE views) will prompt, facilitate and structure discussion. The guide will include questions related to the TDF as this framework will inform the content of training to enable skills development for clinicians and is therefore relevant to understanding how clinicians would use iFraP. The questions will also be informed by the TFA; our overarching theoretical framework of acceptability enabling the investigation of seven constructs of acceptability. Topic guides will be iteratively developed during the study to account for insights not anticipated.

Analysis - Focus groups will be audio-recorded and transcribed. We will use a framework approach to analysis,[48] including a two-stage process to 1) inductively code transcripts followed by 2) a deductive exercise to map identified codes to the domains of relevant theoretical frameworks, including the TDF, TFA, NPT, in accordance with previous research.[49,50] Theoretical frameworks will provide a lens to deepen our understanding of current FLS practice, intervention acceptability, and barriers to, and facilitators of intervention implementation. For example, TDF domains will be mapped to the COM-B model to identify intervention functions and theory-driven behaviour change techniques.[40] Analysis will be undertaken by two members of the study team independently, with a mapping exercise to explore coding consistency. Findings will be discussed with the wider study team and PAG members. Qualitative data will be managed and coded using NVivo 11.

Usual care survey

Participants - an electronic survey (utilising HealthSurvey, hosted by Keele University) will be designed and distributed to nurses or allied health professionals (AHPs) working in UK FLSs, with a target sample size of 80 across the 4 devolved nations. The survey will be distributed by the Royal College of Physicians Fracture Liaison database national audit (FLS-DB), ROS mailing lists, and by researchers to individuals in their known professional networks.

Data collection - Variation in FLS design and specification influences the amount of clinician-patient dialogue about drug recommendations, which has direct implications for implementation of the iFraP intervention and any subsequent trial. For example, the extent of patient contact (e.g. face-to-face, telephone or by letter) will affect the ability to use the CDST within the consultation. The specific content for the usual care survey will be informed by the focus groups, and will aim to describe and quantify the nature and amount of discussion FLSs have with patients about osteoporotic drugs. Any planned changes in service specification will also be explored.

Analysis - Any duplicate entries from the same FLS will be checked for consistency. If there are any differences the respondent will be emailed to clarify. A descriptive analysis will be undertaken. Findings will be discussed with the CoP to discuss implications for iFraP intervention design.

For peer review only - http://bmjopen.bmj.com/site/about/guidelines.xhtml

Results of the descriptive analysis will also be used to create a detailed FLS typology, based on the extent of face-to-face contact to facilitate sampling for the subsequent pilot and feasibility trial.

Step 3: Design and refine the iFraP intervention using field testing
The outputs from studies in Step 1 and 2 will be integrated to inform design of the CDST and training (objective 2, 3b, 3c). iFraP design decision-making will be made and documented by the Study Management Group.[32] Following design of the prototype iFraP consultation intervention (including CDST and training), we will conduct three cycles of in-practice (or field) testing to assess how the fracture prevention components work together, and the acceptability and feasibility of the prototype CDST (objective 3d).

Data integration

Data from the evidence synthesis, Delphi survey and focus groups will be integrated to add rigour, provide greater credibility to results, and generate insights about the intervention in development. Integration will be achieved using a convergent design and merging of the quantitative and qualitative findings using side-by-side joint displays.[51]

A framework for the consultation will be drafted, informed by the consultation stages identified in the evidence synthesis, underpinning theory, frameworks of shared decision-making and principles of health literacy. This will include a series of stages and tasks for the clinician to progress through during the consultation, including gathering information, clinician decision-making, eliciting patient knowledge through to summarising and signposting. Findings from the Delphi analysis (a list of items to be included - and whether they are essential or optional, a list of items excluded, and relevant free-text comments) and qualitative analysis will be mapped to the relevant stage of the consultation in the drafted framework. A separate extraction table will be used for joint display of other contextual considerations for the intervention, derived from the free-text in the Delphi and qualitative findings, and will be organised into considerations with relevance to i) the tool development, ii) the training development and/or iii) subsequent trial design.

The joint display will be interrogated to identify 'meta-inferences'[51]: confirmed, discordant, and expanded findings. Resultant discordant areas, or areas of uncertainty, will be presented to the CoP and PAG for further discussion. Following stakeholder discussion, the list of content and considerations for i) tool development, ii) training development and iii) the trial will be finalised.

iFraP intervention design

Prototype CDST design - The CDST will contain content to support clinician and patient decision-making. The patient-facing component will be designed with the principles of conversation aids, rather than decision aids, to support the discussion between clinician and patient.[52] Frequent stakeholder (CoP and PPIE) workshops throughout iFraP-D will allow members to contribute and provide insight into CDST design. Insights will be considered alongside integration outputs described above in accordance with our informed design approach.[32,39] The prototype tool will be web-based and built using a programmed decision-tree based upon a modified Markov model (a way to model prognosis for clinical problems with ongoing risk). Its development will adhere to international guidance and be informed by a published and process map for DST development.[38] A technical production group will manage the CDST design. A scientific advisory group of osteoporosis academics will advise on the scientific evidence-base that will underpin the CDST. Where possible,

For peer review only - http://bmjopen.bmj.com/site/about/guidelines.xhtml

event rates from existing NICE guidelines and meta-analyses will be utilised. PPIE and clinician members of the CoP will informally test early CDST prototypes.

Training design - Separately, also informed by stakeholder (CoP and PPIE) workshops and integration outputs (e.g. list of content and considerations for iFraP development), a training development group (including input from a behavioural psychologist, patients, expert nurse educator and expert in shared decision-making) will map behaviour change techniques targeted to FLS clinician training needs. Qualitative analysis mapped to the TDF [30] aligned with the COM-B model will support identification of theory-driven behaviour change techniques.[29] This process will allow the training development group to develop an evidence-based clinician training package and associated training manual.

In-practice testing

Design - We will conduct three cycles of in-practice testing of the prototype iFraP consultation in one FLS site. Each of the three cycles will consist of five consultations with patients and post-consultation interviews. At the end of each cycle, the FLS clinician conducting the prototype iFraP consultation will also be interviewed. Each consultation will be audio-recorded, observed, and contain a 'think-aloud' interview

Participants - Patients with a recent fragility fracture (n=15) who are referred for an FLS consultation and clinicians conducting face-to-face FLS consultations (up to n=3). In-practice testing will take place at the Haywood Hospital FLS chosen for its geographical proximity to the study team. The Haywood Hospital FLS operates a one-stop model, meaning that if appropriate, patients have a bone density scan (DXA), nurse assessment, drug treatment recommendation, and blood tests as part of one consultation.

Data collection and procedures - Patients with FLS appointments will be asked to consent to having their consultation observed by a researcher and audio-recorded. For each patient participant, the researcher will conduct a brief (previously developed) warm-up exercise on the 'think-aloud' technique prior to the consultation.[53] In each cycle of consultation and 'think aloud' interview, a three step test interview approach will be used.[54] The topic guide will be developed in collaboration with PAG members.

First, the researcher will observe and listen to the test consultation (noting any visual and verbal cues to be explored in the interview immediately following the consultation) and will make notes using an observation schedule. Patients will be asked to mention (aloud, at the time they arise) any thoughts and feelings, if they feel comfortable to do so, during the consultation as part of a 'think-aloud' process.

Second, patient participants will be interviewed on completion of the consultation using pre-defined and spontaneous probes related to iFraP intervention components, to follow-up issues that emerged during the consultation (e.g. to explore unexpected responses and fill in gaps where the participant may not have commented in real-time). This step will provide evidence on whether risk communication is understood by participants in a consistent way and in the way intended, and how well the intervention is meeting its objectives.

Third, participants will be invited to explain comments made in step 2 and their behaviour during the consultation, and share views and opinions of the consultation. They will be encouraged to say everything they thought about each part of the consultation. Participants will be invited to contact the researcher if any further thoughts come to mind after the interview.

For peer review only - http://bmjopen.bmj.com/site/about/guidelines.xhtml

Post-consultation interviews with clinicians will be conducted as soon after the last consultation in the cycle as possible. A similar three-step approach will be used. In the first step however, these clinician participants will be played audio clips demonstrating where they explained risk, used the CDST and gave recommendations. They will be encouraged to think-aloud and mention any thoughts or feelings during playback. Second and third steps will be as for patient interviews.

Analysis - The framework approach (as described above for focus groups) will be used. The first interviews will be analysed, with help from CoP and PAG members to interpret data. Relevant changes to iFraP will be made ahead of further testing, supported by theoretical frameworks to identify and overcome barriers to implementation, enabling an iterative cycle moving between user feedback and changes to the intervention.

The outcome of this study will finalise a draft iFraP intervention and intervention manual to be tested in a formal pilot and feasibility trial with nested value of information analysis, to evaluate acceptability, feasibility and cost-effectiveness.

ETHICS AND DISSEMINATION

Ethics approval for the work outlined in this protocol was sought and obtained from North West - Greater Manchester West Research Ethics Committee (reference number: 19/NW/0559).

Dissemination and knowledge mobilisation will be facilitated through national bodies and networks such as the ROS, journal papers and conference presentations. The results of this study will be made widely and freely available to all stakeholders; a summary of the results will be published on the Keele University and ROS website. PAG members will advise on how to translate these into easily understandable messages and on how best to disseminate the results to the wider public.

In addition to publications in open-access peer-reviewed journals, we will use NHS networks and links to professional bodies to support dissemination of the findings to all stakeholders and will use social media to promote the findings via our dedicated Twitter and Facebook feeds.

For peer review only

For peer review only - http://bmjopen.bmj.com/site/about/guidelines.xhtml

References

1 National Osteoporosis Society. The Osteoporosis Agenda England: Improving the lives of people with osteoporosis and fragility fractures. 2015.https://theros.org.uk/media/1959/agenda-for-osteoporosis-england-final.pdf

2 NHS RightCare. NHS RightCare scenario: The variation between sub-optimal and optimal pathways. 2017.https://www.england.nhs.uk/rightcare/wp-content/uploads/sites/40/2017/02/rightcare-susans-story-full-narrative.pdf

3 Compston J, Cooper A, Cooper C, *et al.* UK clinical guideline for the prevention and treatment of osteoporosis. *Arch Osteoporos* 2017;**12**:43. doi:10.1007/s11657-017-0324-5

4 National Osteoporosis Society. Effective Secondary Prevention of Fragility Fractures: Clinical Standards for Fracture Liaison Services. 2015.https://theros.org.uk/media/2082/clinical-standards-for-fracture-liaison-services.pdf

5 National Institute for Health and Care Excellence. Bisphosphonates for treating osteoporosis. 2017.https://www.nice.org.uk/guidance/ta464 (accessed 21 Jan 2020).

6 Kanis JA, Svedbom A, Harvey N, *et al.* The Osteoporosis Treatment Gap. *J Bone Miner Res* 2014;**29**:1926–8. doi:10.1002/jbmr.2301

7 Hall SF, Edmonds SW, Lou Y, *et al.* Patient-reported reasons for nonadherence to recommended osteoporosis pharmacotherapy. *J Am Pharm Assoc* 2017;**57**:503–9. doi:10.1016/j.japh.2017.05.003

8 Hiligsmann M, Cornelissen D, Vrijens B, *et al.* Determinants, consequences and potential solutions to poor adherence to anti-osteoporosis treatment: results of an expert group meeting organized by the European Society for Clinical and Economic Aspects of Osteoporosis, Osteoarthritis and Musculoskeletal. *Osteoporos Int* 2019;**30**:2155–65. doi:10.1007/s00198-019-05104-5

9 McHorney CA, Schousboe JT, Cline RR, *et al.* The impact of osteoporosis medication beliefs and side-effect experiences on non-adherence to oral bisphosphonates. *Curr Med Res Opin* 2007;**23**:3137–52.https://www.ncbi.nlm.nih.gov/pubmed/17988435 (accessed 23 Dec 2019).

10 Clark EM, Gould VC, Tobias JH, *et al.* Natural history, reasons for, and impact of low/non-adherence to medications for osteoporosis in a cohort of community-dwelling older women already established on medication: a 2-year follow-up study. *Osteoporos Int* 2016;**27**:579–90. doi:10.1007/s00198-015-3271-2

11 National Osteoporosis Society. Quality Standards for Osteoporosis and Prevention of Fragility Fractures. 2017. https://theros.org.uk/media/0dillsrh/ros-op-standards-november-2017.pdf (accessed 21 Jan 2020).

12 Raybould G, Babatunde O, Evans AL, *et al.* Expressed information needs of patients with osteoporosis and/or fragility fractures: a systematic review. *Arch Osteoporos* 2018;**13**:1–16. doi:10.1007/s11657-018-0470-4

13 Paskins Z, Crawford-Manning F, Cottrell E, *et al.* Acceptability of bisphosphonates among patients, clinicians and managers: A systematic review and framework synthesis. *BMJ Open* 2020;**10**:40634. doi:10.1136/bmjopen-2020-040634

14 Kanis JA, Johnell O, Oden A, *et al.* FRAX^{TM} and the assessment of fracture probability in men and women from the UK. *Osteoporos Int* 2008;**19**:385–97. doi:10.1007/s00198-007-0543-5

For peer review only - http://bmjopen.bmj.com/site/about/guidelines.xhtml

15 Salter C, McDaid L, Bhattacharya D, *et al.* Abandoned Acid? Understanding adherence to bisphosphonate medications for the prevention of osteoporosis among older women: A qualitative longitudinal study. *PLoS One* 2014;**9**. doi:10.1371/journal.pone.0083552

16 Gregson CL, Dennison EM, Compston JE, *et al.* Disease-specific perception of fracture risk and incident fracture rates: GLOW cohort study. *Osteoporos Int* 2014;**25**:85–95. doi:10.1007/s00198-013-2438-y

17 Paskins Z, Jinks C, Mahmood W, *et al.* Public priorities for osteoporosis and fracture research: results from a general population survey. *Arch Osteoporos* 2017;**12**:1–8. doi:10.1007/s11657-017-0340-5

18 Street RL, Makoul G, Arora NK, *et al.* How does communication heal? Pathways linking clinician-patient communication to health outcomes. *Patient Educ Couns* 2009;**74**:295–301. doi:10.1016/j.pec.2008.11.015

19 DiMatteo MR, Sherbourne CD, Hays RD, *et al.* Physicians' Characteristics Influence Patients' Adherence to Medical Treatment: Results From the Medical Outcomes Study. *Heal Psychol* 1993;**12**:93–102. doi:10.1037/0278-6133.12.2.93

20 Stacey D, Légaré F, Lewis K, *et al.* Decision aids for people facing health treatment or screening decisions. *Cochrane Database Syst Rev* 2017;**12**:CD001431. doi:10.1002/14651858.CD001431.pub5

21 Elwyn G, Frosch D, Volandes AE, *et al.* Investing in deliberation: A definition and classification of decision support interventions for people facing difficult health decisions. *Med Decis Mak* 2010;**30**:701–11. doi:10.1177/0272989X10386231

22 McCaffery KJ, Holmes-Rovner M, Smith SK, *et al.* Addressing health literacy in patient decision aids. *BMC Med Inform Decis Mak* 2013;**13**:S10. doi:10.1186/1472-6947-13-S2-S10

23 Paskins Z, Torres-Roldan V, Hawarden A, *et al.* Quality and effectiveness of osteoporosis treatment decision aids: a systematic review and environmental scan. *Osteoporos Int* Published Online First: 2020. doi:10.1007/s00198-020-05479-w

24 Kawamoto K, Houlihan CA, Balas EA, *et al.* Improving clinical practice using clinical decision support systems: A systematic review of trials to identify features critical to success. *Br Med J* 2005;**330**:765–8. doi:10.1136/bmj.38398.500764.8f

25 Garg AX, Adhikari NKJ, McDonald H, *et al.* Effects of computerized clinical decision support systems on practitioner performance and patient outcomes: A systematic review. *J Am Med Assoc* 2005;**293**:1223–38. doi:10.1001/jama.293.10.1223

26 Royal Osteoporosis Society. Effective Secondary Prevention of Fragility Fractures: clinical standards for Fracture Liaison Services. 2019.https://theros.org.uk/media/1eubz33w/ros-clinical-standards-for-fracture-liaison-services-august-2019.pdf (accessed 16 Oct 2020).

27 Horne R, Chapman SCE, Parham R, *et al.* Understanding patients' adherence-related Beliefs about Medicines prescribed for long-term conditions: A meta-analytic review of the Necessity-Concerns Framework. *PLoS One* 2013;**8**. doi:10.1371/journal.pone.0080633

28 Sekhon M, Cartwright M, Francis JJ. Acceptability of healthcare interventions: An overview of reviews and development of a theoretical framework. *BMC Health Serv Res* 2017;**17**:1–13. doi:10.1186/s12913-017-2031-8

29 Michie S, van Stralen MM, West R. The behaviour change wheel: A new method for

For peer review only - http://bmjopen.bmj.com/site/about/guidelines.xhtml

characterising and designing behaviour change interventions. *Implement Sci* 2011;**6**. doi:10.1186/1748-5908-6-42

30 Cane J, O'Connor D, Michie S. Validation of the theoretical domains framework for use in behaviour change and implementation research. *Implement Sci* 2012;**7**:37. doi:10.1186/1748-5908-7-37

31 Murray E, Treweek S, Pope C, *et al.* Normalisation process theory: A framework for developing, evaluating and implementing complex interventions. *BMC Med* 2010;**8**. doi:10.1186/1741-7015-8-63

32 O'Cathain A, Croot L, Duncan E, *et al.* Guidance on how to develop complex interventions to improve health and healthcare. *BMJ Open* 2019;**9**:e029954. doi:10.1136/bmjopen-2019-029954

33 Campbell NC, Murray E, Darbyshire J, *et al.* Designing and evaluating complex interventions to improve health care. *Br Med J* 2007;**334**:455–9. doi:10.1136/bmj.39108.379965.BE

34 Grol R, Wensing M, Eccles M. *Improving patient care: The implementation of change in clinical practice*. Edinburgh: : Butterworth Heinemann. 2005.

35 O'Cathain A, Croot L, Sworn K, *et al.* Taxonomy of approaches to developing interventions to improve health: a systematic methods overview. *Pilot Feasibility Stud* 2019;**5**:1–27. doi:10.1186/s40814-019-0425-6

36 Croot L, O'Cathain A, Sworn K, *et al.* Developing interventions to improve health: A systematic mapping review of international practice between 2015 and 2016. *Pilot Feasibility Stud* 2019;**5**:1–13. doi:10.1186/s40814-019-0512-8

37 Coulter A, Stilwell D, Kryworuchko J, *et al.* A systematic development process for patient decision aids. *BMC Med Inform Decis Mak* 2013;**13**:S2. doi:10.1186/1472-6947-13-S2-S2

38 Elwyn G, Kreuwel I, Durand MA, *et al.* How to develop web-based decision support interventions for patients: A process map. *Patient Educ Couns* 2011;**82**:260–5. doi:10.1016/j.pec.2010.04.034

39 Rousseau N, Turner KM, Duncan E, *et al.* Attending to design when developing complex health interventions: A qualitative interview study with intervention developers and associated stakeholders. *PLoS One* 2019;**14**:1–20. doi:10.1371/journal.pone.0223615

40 Michie S, Atkins LW. *The Behaviour Change Wheel: A Guide to Interventions*. London: : Silverback Publishing 2014.

41 Wenger E. Communities of Practice: Learning, Meaning, and Identity. *Syst Thinker* 1998;**9**:2–3. doi:10.2277/0521663636

42 Staniszewska S, Brett J, Simera I, *et al.* GRIPP2 reporting checklists: Tools to improve reporting of patient and public involvement in research. *BMJ* 2017;**358**:j3453. doi:10.1136/bmj.j3453

43 Grime JC, Ong BN. Constructing osteoarthritis through discourse - A qualitative analysis of six patient information leaflets on osteoarthritis. *BMC Musculoskelet Disord* 2007;**8**:34. doi:10.1186/1471-2474-8-34

44 Brouwers MC, Kho ME, Browman GP, *et al.* AGREE II: Advancing guideline development, reporting and evaluation in health care. *CMAJ* 2010;**182**:E839. doi:10.1503/cmaj.090449

For peer review only - http://bmjopen.bmj.com/site/about/guidelines.xhtml

45 Leventhal H, Benyamini Y, Brownlee S, *et al.* Perceptions of health and illness: Current research and applications. In: Petrie KJ, Weinman J., eds. *Illness representations: Theoretical foundations*. Harwood Academic Publishers. 1997. 19–45.

46 McKenna HP. The Delphi technique: a worthwhile research approach for nursing? *J Adv Nurs* 1994;**19**:1221–5. doi:10.1111/j.1365-2648.1994.tb01207.x

47 Kitzinger J. Qualitative research: introducing focus groups. *BMJ* 1995;**311**:299–302. doi:10.1136/bmj.311.7000.299

48 Ritchie J, Spencer L. Qualitative data analysis for applied policy research. In: Bryman A, Burgess RG, eds. *Analysing qualitative data*. London: : Routledge 1994. 173–94.

49 Saunders B, Hill JC, Foster NE, *et al.* Stratified primary care versus non-stratified care for musculoskeletal pain: Qualitative findings from the STarT MSK feasibility and pilot cluster randomized controlled trial. *BMC Fam Pract* 2020;**21**:1–12. doi:10.1186/s12875-020-1098-1

50 Saunders B, Bartlam B, Foster NE, *et al.* General Practitioners' and patients' perceptions towards stratified care: A theory informed investigation. *BMC Fam Pract* 2016;**17**:125. doi:10.1186/s12875-016-0511-2

51 Younas A, Pedersen M, Durante A. Characteristics of joint displays illustrating data integration in mixed-methods nursing studies. *J Adv Nurs* 2020;**76**:676–86. doi:10.1111/jan.14264

52 Kunneman M, Montori VM, Castaneda-Guarderas A, *et al.* What Is Shared Decision Making? (and What It Is Not). *Acad Emerg Med* 2016;**23**:1320–4. doi:10.1111/acem.13065

53 Beatty P, Willis GB, Beatty PC. Research synthesis: The practice of cognitive interviewing. *Public Opin Q* 2007;**71**:287–311. doi:10.1093/poq/nfm006

54 Paap MCS, Lange L, van der Palen J, *et al.* Using the Three-Step Test Interview to understand how patients perceive the St. George's Respiratory Questionnaire for COPD patients (SGRQ-C). *Qual Life Res* 2016;**25**:1561–70. doi:10.1007/s11136-015-1192-3

For peer review only

Author's contributions

ZP conceived the study. ZP, LB, EC, JF, SL, JJE, EMC, ST, SC, CG, CPI, TON, CM and CJ reviewed the study design and contributed to study implementation as part of the iFraP Study Management Group. JP and RH contributed to study design as expert study advisors. FCM will support study set up and data collection. JL, SL, and SR will form the iFraP training development group. ST and SC will inform design and development of the prototype iFraP CDST. CPI will provide health economic expertise. All authors contributed to refinement of the study protocol and approved the final manuscript.

Funding

This study was funded by the National Institute for Health Research (NIHR) [Clinician Scientist Award (CS-2018-18-ST2-010)/NIHR Academy], the Royal Osteoporosis Society (REF: 430), and the Haywood Rheumatology Research and Development Foundation (not applicable).

The views expressed are those of the author(s) and not necessarily those of the National Health Service, the NIHR, or the Department of Health and Social Care.

Word count: 5649

Exclusive Licence

I, the Submitting Author has the right to grant and does grant on behalf of all authors of the Work (as defined in the below author licence), an exclusive licence and/or a non-exclusive licence for contributions from authors who are: i) UK Crown employees; ii) where BMJ has agreed a CC-BY licence shall apply, and/or iii) in accordance with the terms applicable for US Federal Government officers or employees acting as part of their official duties; on a worldwide, perpetual, irrevocable, royalty-free basis to BMJ Publishing Group Ltd ("BMJ") its licensees and where the relevant Journal is co-owned by BMJ to the co-owners of the Journal, to publish the Work in BMJ Open and any other BMJ products and to exploit all rights, as set out in our licence.

The Submitting Author accepts and understands that any supply made under these terms is made by BMJ to the Submitting Author unless you are acting as an employee on behalf of your employer or a postgraduate student of an affiliated institution which is paying any applicable article publishing charge ("APC") for Open Access articles. Where the Submitting Author wishes to make the Work available on an Open Access basis (and intends to pay the relevant APC), the terms of reuse of such Open Access shall be governed by a Creative Commons licence – details of these licences and which Creative Commons licence will apply to this Work are set out in our licence referred to above.

Figures (uploaded separately)

Fig 1**.** iFraP-D Logic Model

Fig 2**.** Inter-relation between the implementation of change model and iFraP-D data collection methods

Competing interests

For peer review only

Dr Zoe Paskins reports grants from the NIHR Clinician Scientist Award (CS-2018-18-ST2-010), Royal Osteoporosis Society, and the Haywood Rheumatology Research and Development Foundation to conduct this study. Prof Clare Jinks reports funding from the NIHR Applied Research Collaboration (ARC) West Midlands; Prof Christian Mallen reports grants from the NIHR Research Professorship in General Practice (NIHR-RP-2014–04–026), the NIHR School for Primary Care Research and NIHR Applied Research Collaborations (West Midlands), Wellcome, Medical Research Council, Dunhill, Versus Arthritis and Bristol Myer-Squibb, outside the submitted work; Dr Fay Crawford-Manning reports grants from NIHR Clinical Research Network Scholar Programme; Simon Thomas and Prof Stephen Chapman report receiving funds from National Institute for Health Research (NIHR) [Clinician Scientist Award (CS-2018-18-ST2-010)/NIHR Academy] and the Royal Osteoporosis Society via Prescribing Decision Support Ltd to support the development of the iFraP decision support tool; Prof. Terence O'Neill reports grants and non-financial support from AMGEN and the NIHR Manchester Biomedical Research Centre outside the submitted work; Dr Cynthia Iglesias reports grants from NIHR during the conduct of the study and disclosed being a member of NICE's Medical Technologies Advisory Committee outside the submitted work; Dr John Edwards reports grants as a NIHR Academic Clinical Lecturer in Primary Care (CL-2016-10-003). Dr Emma M Clark reports grants from Versus Arthritis, NIHR RfPB, HQIP and the Royal Osteoporosis Society. Dr Laurna Bullock, Dr Elizabeth Cottrell, Prof Robert Horne, Prof Janet Lefroy, Prof Chris Gidlow, Dr Jane Fleming, Prof Sarah Ryan, and Sarah Leyland have nothing to disclose.

For peer review only - http://bmjopen.bmj.com/site/about/guidelines.xhtml

Problem: Poor fracture prevention treatment uptake

Targeted determinants: patient health beliefs

Aim: to improve informed decision making in FLS consultations

Intervention Resources

- FLS service
- Evidence summaries
- CDST to depict individualized fracture risk and treatment benefits/harms
- Training package
- Individualised patient information leaflets
- Patient information website

→

Processes

- FLS HCP receives training in using CDST communicating risk, health literacy approaches, eliciting and addressing patient preferences and values and SDM
- Trained HCP conducts FLS consultation using CDST
- Patient given ind. info. leaflet and signposted to website
- GP receives same information

→

Outputs

FLS HCP

- Increased confidence in using CDST, communicating risk, health literacy approaches, eliciting and addressing patient preferences and values and SDM

Post-consultation

- Consistency of messages across primary care and FLS

→

Short term outcomes

CLINICIAN

- Identifies appropriate treatment options

PATIENT

- Increased understanding of own fracture risk & risk/benefit of treatments
- Increased self-efficacy & enablement
- Trust in clinician

↓

- Reduced decisional conflict
- Improved decision quality
- Increased rates of treatment initiation

→

Long term outcomes

PATIENT

- Persistence with fracture prevention treatments at 12 months
- Lifestyle outcomes (PA, smoking, alcohol)
- *Increased incidence of drug adverse events*
- Fracture reduction

NHS

- Guideline adherence
- Reduced costs of fracture care

Context: Consultations conducted in pre-existing specialist face-to-face fracture prevention services (Fracture Liaison Service - FLS)
Contextual factors: Poor uptake of NICE/NOGG guidelines; disconnect in advice given to patient between FLS and primary care; media and wider social influences on health behaviours

**CDST computerised decision support tool, FLS Fracture Liaison Service, HCP healthcare professional, PA physical activity, GP general practitioner, SDM shared decision-making*

For peer review only - http://bmjopen.bmj.com/site/about/guidelines.xhtml

Steps 1 – 3 of iFraP intervention development

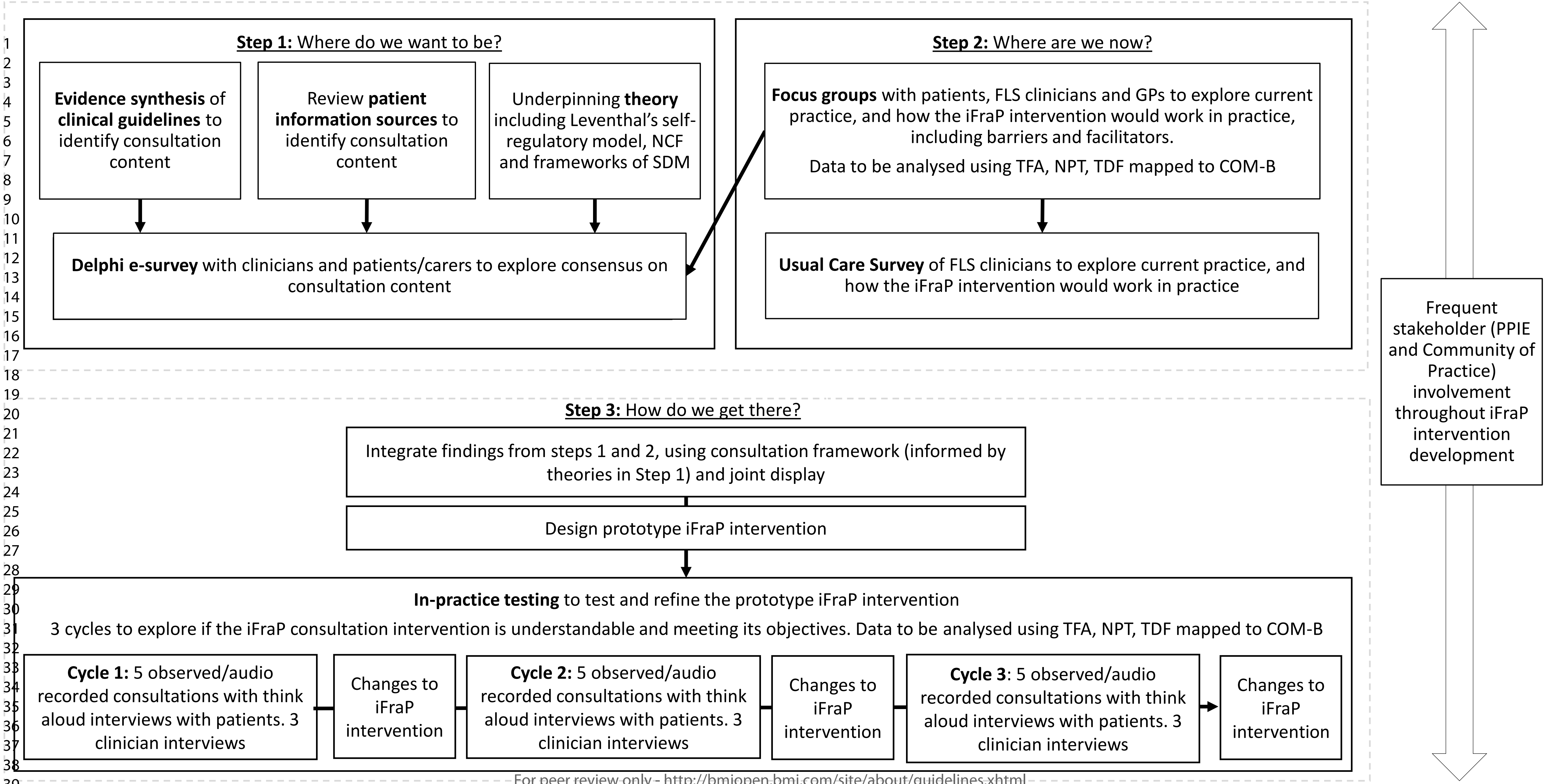

NCF necessity concerns framework; TDF theoretical domains framework; TFA theoretical framework of acceptability; NPT normalisation process theory; SDM shared decision-making; PPIE patient and public engagement and involvement; FLS Fracture Liaison Service; GP general practitioner

Supplementary Table 1. Eligibility criteria for osteoporosis guideline evidence synthesis

**Inclusion criteria**	
**Population**	Patients at risk of osteoporosis or with fragility fractures
**Interventions**	Clinician-patient consultations to assess risk and initiate/recommend treatment for fracture prevention
**Outcomes**	Assessment<br>Explanations<br>Decision-making
**Setting**	Relevant to UK primary or secondary care (e.g. UK, European or international)
**Date**	Those guidelines that have been developed, reviewed or revised within the past 10 years will be used (2009 to Feb 2019)
**Exclusion criteria**	
Guidelines relating to:<br>• managing patients already on fracture prevention treatments<br>• disease specific conditions (e.g. screening for osteoporosis in inflammatory bowel disease)<br>• patients with one specific fracture site (e.g. hip fracture)<br>• steroid-induced osteoporosis<br>• specific countries other than UK<br>• fracture assessment and management<br>Guidelines related to pre-clinical and animal studies<br>Withdrawn guidance<br>The original research manuscripts underpinning the guidelines<br>Studies not in the English language.	

For peer review only
